# Characterization and risk stratification of coronary artery disease in people living with HIV: a global systematic review

**DOI:** 10.3389/fcvm.2025.1586019

**Published:** 2025-08-12

**Authors:** Martins Nweke, Sam Ibeneme, Julian D. Pillay, Nombeko Mshunqane

**Affiliations:** ^1^ Department of Physiotherapy, Faculty of Health Sciences, University of Pretoria, Pretoria, South Africa; ^2^ Global Health and Sustainability, Faculty of Health Sciences, Durban University of Technology, Durban, South Africa; ^3^ Department of Physiotherapy, David Umahi Federal University of Health Sciences, Uburu, Ebonyi, Nigeria

**Keywords:** coronary artery disease, HIV, risk stratification, epidemiological model, systematic review

## Abstract

**Background:**

Coronary artery disease (CAD) is a leading cause of mortality among people living with HIV (PLWH). Risk stratification remains inconsistent due to geographic disparities, ART-related metabolic effects, and overreliance on strength of association. This review synthesizes global evidence to classify CAD risk factors in PLWH, aiming to improve predictive models and preventative strategies.

**Methods:**

Following the PRISMA 2020 guidelines, a systematic review was conducted across six databases: PubMed, Scopus, Web of Science, Medline, CINAHL, and African Journals (SABINET). Two independent reviewers screened studies and extracted data. Narrative synthesis and meta-analysis were conducted. Risk factors were classified using Rw, causality index (CI), and public health priority (PHP).

**Findings:**

Twenty-two studies involving 103,370 participants were included. First-class risk factors (CI: 7–10) included hypertension (OR: 4.9; *p* < 0.05; Rw: 4.5), advanced age (≥50 years) (OR: 4.96, *p* < 0.05, Rw: 3.58), dyslipidemia (OR: 2.15, *p* < 0.04, Rw: 2.15), and overweight/obesity (OR: 1.81, *p* < 0.05, Rw: 1.36). Second-class risk factors (CI: 5–6) included family history of CVD (OR: 3.25, *p* < 0.05; Rw: 2. 24). Third-class risk factors (CI ≤4) included diabetes (OR: 2.64, *p* < 0.05, Rw: 1.32), antiretroviral therapy exposure (OR: 1.68, *p* < 0.05, Rw: 0.63), and homosexuality (OR: 1.82, *p* < 0.05, Rw: 0.62). Critical thresholds (cumulative Rw: 14.8 and 8.0) were set at 75th and 50th percentiles of cumulative Rw. At GTT value of 0.50, the parsimonious global clinical prediction model for HIV-related CAD included age, hypertension, dyslipidemia, family history of CVD, diabetes, and overweight/obesity (Rw: 15.5, GTT: 4.05). For primary prevention, the optimal model comprised hypertension, dyslipidemia, and obesity (Rw: 8.01, GTT: 2.07). Advanced age and hypertension were “necessary causes” of CAD among PLWH.

**Conclusion:**

Association strength alone cannot determine CAD risk. Cumulative risk indexing and responsiveness provide a robust framework. Prevention should prioritize hypertension and dyslipidemia management, with interventions for obesity, smoking, and virological failure. Age and hypertension should prompt cardiovascular screening. Standardized risk definitions, accounting for the role of protective factors and integrating evidence with domain knowledge are vital for improved CAD risk stratification and prediction in PLWH. Routine cardiovascular screening in HIV care remains essential.

**Systematic Review Registration:**

https://www.crd.york.ac.uk/PROSPERO/view/CRD42024524494, PROSPERO CRD42024524494.

## 1 Introduction

Antiretroviral therapy (ART) has transformed human immunodeficiency virus (HIV) from a fatal disease to a chronic condition, markedly improving life expectancy (1). This shift has increased the burden of non-communicable diseases, including cardiovascular disease (CVD), among people living with HIV (PLWH) (2). Coronary artery disease (CAD) is a leading cause of morbidity and mortality in PLWH, with higher incidence rates than in HIV-negative individuals (3). Despite extensive research, gaps persist in our understanding of CAD-specific risk factors, their causal interactions, and geographic variability.

CAD in PLWH arises from a complex interplay between traditional cardiovascular risk factors and HIV-specific mechanisms. Traditional risk factors, such as hypertension, diabetes, dyslipidemia, smoking, obesity, and physical inactivity, contribute to atherosclerosis and cardiovascular complications in both HIV-positive and HIV-negative populations. However, PLWH also experience chronic systemic inflammation, immune activation, ART-induced metabolic disturbances (e.g., dyslipidemia and insulin resistance), and direct viral effects on vascular endothelial function (4, 5). While some studies emphasize immune dysregulation in CAD pathogenesis, others highlight ART-related metabolic disturbances, particularly prolonged exposure (6, 7). These divergent findings indicate the need for a comprehensive synthesis of the traditional and HIV-specific risk factors across diverse populations.

Most research has been conducted in high-income settings, despite low- and middle-income countries (LMICs) having the highest HIV burden (8, 9). Geographic disparities in ART regimens, healthcare access, and socio-economic conditions influence CAD risk profiles (10, 11). In high-income countries, research often centers on ART-induced metabolic changes, whereas studies from LMICs highlight untreated hypertension, lifestyle-related factors, and limited access to health care (12, 13). Additionally, methodological inconsistencies, such as variation in cohort characteristics, statistical modeling, and CAD endpoint definitions, complicate direct comparisons across studies (14, 15). Addressing these limitations requires a global, systematic synthesis using structured epidemiological frameworks to enhance CAD risk stratification in PLWH.

This review applied four complementary epidemiological frameworks to provide a structured, theory-informed evaluation of CAD risk factors in PLWH. The epidemiological triangle contextualizes disease as an interaction between the agent (HIV and ART), host (genetics, comorbidities), and environment (socioeconomic determinants, healthcare access) (16). Bradford Hill's criteria assess the strength and consistency of causal associations (17), while Rothman's causal pie model identifies necessary, component, and sufficient causes also known as primary risk factors, contributory risk factors and causal path, respectively (18). Nweke's cumulative risk index refines traditional risk stratification approaches by incorporating predictive consistency, irreversibility, and temporality (19).

While each model has limitations, such as the subjectivity of Bradford Hill's criteria (17) and Rothman's limited incorporation of social determinants, integrating them offers a robust framework for stratifying CAD risk in PLWH.

Through the systematic classification of CAD risk factors based on predictive strength and causal relevance, this review aims to improve risk prediction and guide resource allocation, particularly in LMICs. A structured risk stratification approach enables clinicians and public health professionals to identify causal pathways and optimize preventive strategies. To guide this review, we posed the following research question: What are the key cardiovascular, HIV-related, intrinsic, and extrinsic risk factors for CAD in PLWH, and how can epidemiological models be applied to classify and stratify these risks for improved prediction and prevention?

To address this, the study was guided by the following objectives.

1. Identify and classify the primary risk factors associated with CAD in PLWH, including cardiovascular, HIV-related, intrinsic, and extrinsic factors.
2. Stratify the risk factors based on their predictive strength; they were categorized into “necessary causes” (e.g., smoking, viral load, HCV) and “synergistic component causes” (e.g., socioeconomic factors, ART use) using a causality index (CI).
3. Stratify risk factors based on public health priority (PHP)
4. Determine the implications of CAD risk stratification for developing predictive and preventive models tailored to PLWH, enabling more accurate clinical risk assessment and targeted public health interventions.

## 2 Methods

### 2.1 Study design

This systematic review followed the Preferred Reporting Items for Systematic Reviews and Meta-Analyses (PRISMA) guidelines to ensure methodological rigor, transparency, and reproducibility (20). The review was registered with PROSPERO (CRD42024524494).

### 2.2 Conceptual and theoretical underpinning

To address the complexity of CAD risk among PLWH, we applied four theoretical frameworks: epidemiological triangle, Bradford Hill's criteria, Rothman's causal pie model, and Nweke's cumulative risk index. These models were selected for their strengths in capturing multifactorial disease causation and addressing the limitations of individual theories. The specific applications and limitations are detailed in Supplementary File 1.

These epidemiological models guided both data the extraction and risk stratification in this systematic review. Specifically:

i. The epidemiological triangle informs the broad sampling of risk factors, ensuring that the agent (HIV/ART), host (individual factors), and environmental influences (socioeconomic factors and healthcare access) are captured holistically.
ii. Bradford Hill's criteria informed the assessment of causality.
iii. Rothman's causal pie model allowed the classification of necessary, component, and sufficient risk “causes.”
iv. Nweke's cumulative risk index refines the estimation of cumulative risk based on Hill's criteria, CI, critical risk threshold, and PHP.

### 2.3 PECOT criteria

We used the Population, Exposure, Comparison, Outcome, and Timeframe (PECOT) framework to define our inclusion criteria.

**Population (P)**: PLWH of any age, sex, or geographic location, with or without CAD.

**Exposure (E)**:

1. **Agent-related factors**: HIV duration, ART type, ART duration, CD4 count, and viral load.
2. **Cardiovascular risk factors**: Hypertension, diabetes, dyslipidemia, obesity, smoking, and prior cardiovascular events.
3. **Sociodemographic factors**: Age, sex, income, education level, healthcare access, and lifestyle factors such as diet and physical activity.

**Comparison (C):** PLWH with and without CAD. Studies that provided risk estimates comparing different exposure levels (e.g., ART-exposed vs. ART-naïve) were also included.

**Outcome (O):** Clinically confirmed CAD, defined as any of the following:

i. Acute myocardial infarction
ii. Angina (stable or unstable)
iii. Coronary atherosclerosis confirmed by imaging or clinical diagnosis
iv. Ischemic heart disease-related mortality

**Timeframe (T):** Longitudinal studies, case-control studies, and meta-analyses conducted from database inception to January 2024. Only studies with clearly defined follow-up periods were included to ensure robust temporal associations between exposure and CAD risk.

### 2.4 Inclusion and exclusion criteria

#### 2.4.1 Inclusion criteria

**Population:** PLWH with or without CAD; all ages, sexes, and geographical locations.

**Study design:** Cohort studies (prospective and retrospective), case-control studies, meta-analyses and systematic reviews that provided pooled risk estimates, and nested cross-sectional studies.

**Exposure and risk factors:** Studies assessing traditional cardiovascular risk factors (hypertension, diabetes, dyslipidemia, obesity, smoking, and physical inactivity). Studies examining HIV-related factors (ART use, HIV duration, immune function [CD4 count, viral load], and inflammation. Studies analyzing sociodemographic and environmental influences (income, education, healthcare access, and air pollution).

**Outcomes:** Studies reporting clinically confirmed CAD (acute myocardial infarction, angina, ischemic heart disease, or atherosclerosis). Studies that include risk estimates (odds ratio, hazard ratio, or relative risk) or data are sufficient to compute risk estimates.

**Timeframe:** Studies published up to January 2024.

**Language:** Irrespective of the language. Articles published in languages other than English were translated using Google Translate.

#### 2.4.2 Exclusion criteria

**Study design:** Simple cross-sectional studies, qualitative studies, editorials, case reports, conference abstracts, and commentaries.

**Population:** Studies that combined HIV-positive and HIV-negative populations and did not separately report CAD risk for PLWH.

**Outcomes:** Studies reporting only the prevalence or frequency of CAD risk factors did not include a measure of association (e.g., odds ratio or hazard ratio). Studies that assessed general CVD outcomes but presented no specific report on CAD.

**Bias and data quality:** Studies with a high risk of bias [assessed using the Joanna Briggs Institute (JBI) risk-of-bias tool]. Studies with incomplete or non-reproducible data**.**

### 2.5 Outcome definitions and measurements

#### 2.5.1 Primary outcomes

The primary outcomes included the risk factors associated with CAD in PLWH. The risk factors were categorized into four domains.

**HIV-specific risk factors:** HIV duration, ART type and duration of ART, and CD4 count and viral load.

**Cardiovascular risk factors:** hypertension (systolic blood pressure ≥140 mmHg and/or diastolic blood pressure ≥90 mmHg), diabetes mellitus (fasting glucose ≥126 mg/dl or HbA1c ≥ 6.5%), dyslipidemia (LDL cholesterol ≥130 mg/dl or total cholesterol ≥200 mg/dl), obesity [body mass index (BMI) ≥ 30 kg/m^2^], smoking [current or past tobacco use (self-reported or biochemical validation)], and a history of cardiovascular events (e.g., acute myocardial infarction and ischemic heart disease). CAD was diagnosed on the basis of electrocardiographic abnormalities, imaging confirmed atherosclerosis, and clinically confirmed ischemic heart disease.

**Sociodemographic factors:** age, sex, genetic predispositions, level of education, income and employment status, healthcare accessibility, and environmental factors (e.g., diet and air pollution).

#### 2.5.2 Secondary outcomes

From the above data, we calculated the temporality of exposure-outcome relationships (assessed whether CAD risk factors preceded disease onset (21, 22). Consistency of associations across studies (evaluated using Nweke's cumulative risk index) (21–24). CI was estimated based on Bradford Hill's criteria, Rothman's causal pie model, and an emerging hypothesis (22). Nature/stage of factors in terms of bio-behavioral status (22). We also assessed PHP (22).

### 2.6 Search strategy

We searched multiple electronic databases to ensure broad coverage of the relevant studies. The databases searched included PubMed/MEDLINE, SCOPUS, EMBASE, Cochrane Library, Web of Science, Cumulative Index for Nursing and Allied Health Literature (CINAHL), and African Journals Online (AJOL)/SABINET.

The reference lists of the included studies and relevant systematic reviews were manually searched for additional articles. Gray literature sources, including conference proceedings and preprint repositories, were considered to minimize publication bias.

This search strategy was developed in collaboration with an experienced information specialist. The search terms were based on Medical Subject Headings (MeSH) and free-text keywords. The search strategy was initially tested in PubMed and adapted for each database using appropriate Boolean operators (AND, OR, NOT) and truncation symbols, where applicable.

### 2.7 Study selection

Search results were exported to EndNote 20 for deduplication and reference management. Two independent reviewers screened the titles and abstracts by considering the predefined inclusion and exclusion criteria. If the studies were selected for full-text screening, full texts were retrieved and assessed for inclusion. Two independent reviewers conducted the selection process and resolved any disagreements through discussion or consultation with a third reviewer.

**Figure 1:**
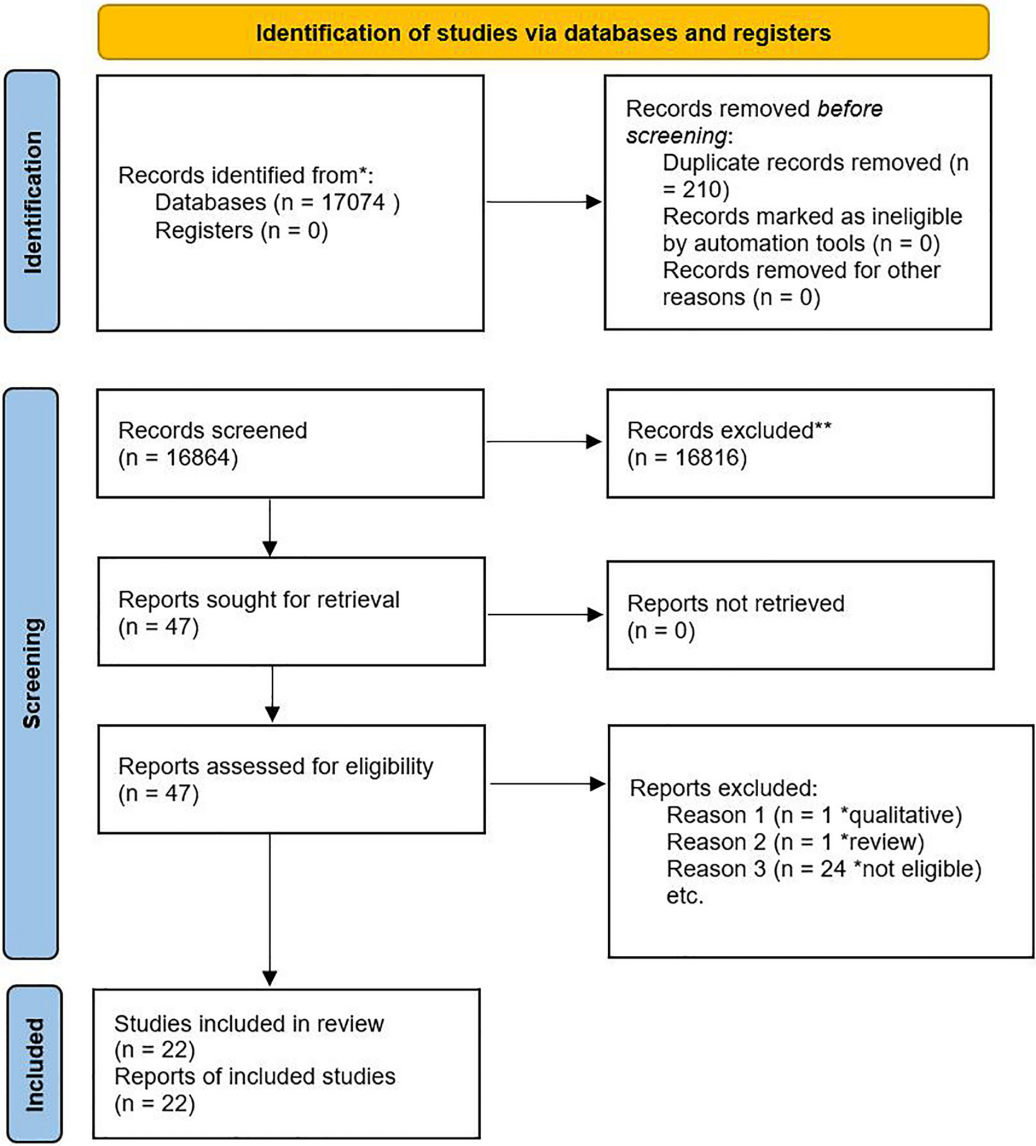
PRISMA flow diagram showing the selection process.

Reasons for exclusion were systematically documented. The selection process was represented using the PRISMA flow diagram (Figure 1).

### 2.8 Data extraction

Data were extracted using a standardized data extraction template to capture study characteristics, exposure variables, and outcome measures. Two reviewers independently extracted the data, and discrepancies were resolved through discussion or third-party arbitration.

### 2.9 Quality control and data validation

Quality control measures were applied throughout the extraction process to minimize bias and enhance data reliability. The extracted data were crosschecked to ensure consistency and accuracy. When multiple studies reported overlapping populations, they were carefully evaluated to ensure nonredundant data extraction. This structured data extraction approach ensures data integrity, comparability, and reproducibility in accordance with PRISMA recommendations.

### 2.10 Risk of bias assessment

The methodological quality of the included studies was assessed using the JBI risk-of-bias assessment tool for case-control and cohort studies (25). This tool systematically evaluates the study design, sampling, measurement, and analytical approaches to determine the potential risk of bias in each study. Two independent reviewers conducted assessments to ensure reliability and minimize subjectivity. Discrepancies were resolved by discussion or consultation with a third reviewer. Based on the JBI criteria, each study was classified as having a low, moderate, or high risk of bias (Supplementary File 3).

Studies with a high risk of bias were interpreted with caution and considered for sensitivity analysis. Studies with critical methodological flaws that could not be adjusted for were excluded from the final analysis.

### 2.11 Data analysis

We present the study characteristics using an evidence table (Table 1). The strength of association between risk each factor and CAD in PLWH was quantified using odds ratios (ORs), relative risks (RRs), and hazard ratios (HRs). These effect sizes were extracted from the included studies to assess the magnitude of the CAD risk associated with different exposures. Where possible, 95% confidence intervals (CIs) were used to estimate the precision of each measure. To ensure consistent interpretation of the risk estimates, OR/RR/HR >1.0 indicated a positive association between exposure and CAD risk; OR/RR/HR <1.0 suggested a protective effect, and OR/RR/HR = 1.0, implying no association between exposure and CAD.

**Table 1:** Study and demographic characteristics.

Author (year)	Definition of coronary artery disease (CAD)	Age	Sex (% female)	Race/Ethnicity	Design	Methods	Sample size	Follow-up	Setting	Country (continent)
Berquist et al. (38)	Self-reported angina, myocardial infarction, or coronary revascularization	52.0 (43.0–60.0)	36.1	–	Case-control	Retrospective	70 (24 cases, 46 controls)	–	Alfred Hospital, Melbourne	Australia (Australia)
Bucher et al. (45)	Myocardial infarction, unstable angina, coronary artery bypass grafting, angioplasty/stenting, or fatal coronary event	≥16	21.4	93.3	Case-control	Prospective	490 (98 cases, 392 controls)	–	Laboratory of the Institute for Lipid Metabolism	Germany (Europe)
Chammartin et al. (42)	Myocardial infarction, coronary angioplasty/stenting, or coronary artery bypass grafting	<50 ≥65	28.1	–	Cohort	Prospective	9,259 (199 cases, 8,960 controls)	11.1 (5.2–18.1)	Clinics	Switzerland (Europe)
Dale et al. (34)	Coronary heart disease (Framingham Risk Scores)	48.3 (8.9)	100	5.7	Cohort	Prospective	53	1–17	Community	USA (North America)
Egaña-Gorroño et al. (46)	Acute myocardial infarction or related hospital events in HIV patients	44 (12)	11.1	94.4	Case-control	Retrospective	72 (18 cases, 54 controls)	–	Hospital Clinical of Barcelona	Spain (Europe)
Engel et al. (43)	History of myocardial infarction, angina, or coronary revascularization	54 (−)	13.8	–	Case-control	Prospective	1,078 (333 cases, 745 controls)	–	Clinics	Switzerland (Europe)
Escaut et al. (47)	Angina pectoris, unstable angina, or acute myocardial infarction in HIV-infected patients.	44.3 (7.65)	21.4	93.3	Cohort	Prospective	840 (17 cases, 823 controls)	7.0	Clinics	France (Europe)
Kaplan et al. (33)	10-year risk of myocardial infarction, CAD death, unstable angina, or chronic angina	43.5 (9.7)	50.0	36.8	Nested cross-sectional	Prospective	208	–	Community	USA (North America)
Lai et al. (36)	Silent CAD (≥50% coronary stenosis)	44.0 (39.9–46.8)	35.8	90.9	Cohort	Prospective	165 (24 cases, 141 controls)	–	HIV clinic	USA (North America)
Freiberg et al. (35)	Myocardial infarction, coronary artery bypass grafting, and percutaneous coronary intervention	47.8 (10/7)	0.0	47.5	Cohort	Retrospective	2,425 (738 cases, 1,687 controls)	7.3	Database/registry	USA (North America)
Fuchs et al. (48)	Myocardial infarction, angina, or coronary intervention	39.0 (9.9)	40.9	66.0	Nested cross-sectional	Prospective	3,829	–	Public outpatient centers	Brazil (South America)
Hadigan et al. (37)	CAD defined via coronary artery calcium scoring as a marker of subclinical atherosclerosis in HIV-infected women.	43.5 (0.7)	–	–	Cohort	Prospective	364 (91 cases, 273 controls)	–	Clinical Research Center of the Massachusetts Institute of Technology	USA (North America)
Longenecker et al. (49)	CAD was defined as Segment involvement score (SIS) > 0; severe CAD (SSS >3).	57 (53.0–62.0)	63.0	–	Cohort	Prospective	200 (100 cases, 100 controls)	2	The Joint Clinical Research Centre in Kampala	Uganda (Africa)
Lui et al. (50)	CAD confirmed by clinical or imaging	53.7 (9.5)	11.3	–	Cohort	Prospective	115 cases, 71 controls	–	The Prince of Wales Hospital Infectious Diseases Clinic	Hong Kong (Asia)
May et al. (44)	Myocardial infarction and angina, coronary revascularization	50.0 (46.0–56.0)	0	–	Secondary analysis of cohort studies	Retrospective	13,100	8.7	–	Switzerland (Europe)
Mushin et al. (39)	Mycardial infarction, ischemic heart disease, angiogram-confirmed CAD or coronary artery bypass grafting.	53 (46.0–60.0)	–	–	Case-control	Retrospective	477 (160 cases, 317 controls)	–	The Alfred Hospital, Melbourne	Australia (Australia)
Pullinger et al. (51)	CHD risk estimated using Framingham Risk Score	45.3 (8.3)	26.7	42.6	Nested cross-sectional	Prospective	267 (80 cases, 187 controls)	–	Clinical Research Center	USA (North America)
Worm et al. (32)	Myocardial infarction, coronary intervention, or CAD-related death.	48.5 (41.0–55.3)	26.0	–	Cohort	Prospective	33,347	1,59,971 person years	Outpatient clinics	Europe, Australia and the US
Urina-Jassir et al. (52)	Coronary stenosis confirmed on imaging or myocardial infarction or ischemic heart disease.	<50 ≥ 50	14.0	–	Cohort	Prospective	36,483	–	Hospital setting	Columbia (South America)
Trøseid et al. (53)	Obstructive CAD: ≥ 50% stenosis; Nonobstructive CAD: 1%–49% stenosis; No CAD: 0% stenosis	54.8 (47.0–61.3)	11.8	–	Nested cross-sectional	Prospective	254 (140 cases, 114 controls)	–	–	Norway (Europe)
Trevillyan et al. (40)	Acute myocardial infarction, positive coronary angiogram, or clinical diagnosis of CAD.	52.3 (42–61)	10.6	–	Case-control	Retrospective	70 (24 cases, 46 controls)		Clinic	Australia
Trevillyan et al. (41)	CAD defined based on documented history of myocardial infarction, angiographically confirmed coronary disease, or revascularization	50.0 (26.0–71.0)	5.9	–	Case-control	Retrospective	204 (136 cases, 68 controls)	7,927 patient years	The Alfred Hospital, Melbourne	Australia

To evaluate the clinical and epidemiological relevance of the findings, the thresholds established by Chen et al. (26) were applied, where OR/RR/HR ≈ 1.0–1.5 indicated a minimal or small association, OR/RR/HR ≈ 1.5–3.0 indicated a moderate association, OR/RR/HR ≥3.0–4.0 indicated a strong association, and OR/RR/HR ≥10 indicated a very strong association.

To enhance comparability, risk estimates were transformed into standardized effect sizes where possible. Additionally, Nweke's CI was employed to integrate both statistical significance (strength of association) and predictive consistency, refining the ranking of CAD risk factors in PLWH (19). The summarized effect sizes and their confidence intervals are presented in Table 2 and categorized by risk factor type.

**Table 2:** Result of individual studies showing factors associated with CAD in PLWH.

Study	Ref category	Effect size	Lower limit	Upper limit	Effect size type	Adjusted (yes/no)	Study design	Country	Continent
ART exposure									
Use of protease inhibitors									
Berquist et al., 2017 (38)	Yes	19.95*	1.01	383.28	OR	Yes	Case-control	Australia	Australia
Lai et al. 2008 (36)	Yes	2.84*	1.11	7.27	OR	No	Cohort	Europe, Australia, US	Europe & America
Worm et al., 2009 (32)	Yes	2.00*	1.55	2.59	OR	No	Cohort	Switzerland	Europe
Chammartin et al., 2022 (42)	Yes	1.57*	1.14	2.16	HR	Yes	Case-control	Barcelona	Europe
Egana-Gorrono et al., 2012 (46)	Yes	8.68	0.46	12.87	OR	No	Case-control	Switzerland	Europe
Escaut et al., 2003 (47)	Yes	1.53*	0.56	4.173	OR	No	Cohort	France	Europe
Kaplan et al., 2007 (33)	Yes	1.35*	0.97	1.84	OR	No	Cohort	USA	North America
Trevillyan et al. 2013 (41)	Yes	0.94	0.50	1.76	OR	No	Matched case-control	Australia	Australia
Trevillyan et al. 2017 (40)	Yes	0.81	0.34	2.0	OR	No	Matched case-control	Australia	Australia
Abacavir use/exposure									
Berquist et al., 2017 (38)	Yes	5.12*	1.70	15.39	OR	No	Case-control	Australia	Australia
Bucher et al. 2012 (45)	–	1.19*	1.05	1.35	OR	No	Case-control	Switzerland	Europe
Chammartin et al. 2022 (42)	Yes	1.83*	1.31	2.55	HR	Yes	Cohort	Switzerland	Europe
Engel et al. 2021 (43)	Yes	1.82*	1.27	2.59	OR	Yes	Case-control	Switzerland	Europe
Mushin et al. 2023 (39)	Yes	1.87*	1.14	3.07	OR	No	Case-control	Australia	Australia
Trøseid et al. 2024 (53)	Yes	0.8149	0.394	1.6854	OR	No	Nested cross-sectional	Norway	Europe
Trevillyan et al. 2013 (41)	Yes	2.10	0.056	1.608	OR	No	Matched case-control	Australia	Australia
NRTI exposure									
Lai et al. 2008 (36)	Yes (≥6 months)	2.20	0.82	5.85	OR	No	Cohort	USA	North America
Worm et al., 2009 (32)	Yes	2.51*	1.79*	3.52*	OR	No	Cohort	Europe, Australia, US	Europe & America
NNRTI exposure									
Lai et al. 2008 (36)	Yes (≥6 months)	3.42*	1.03	10.6	OR	No	Cohort	USA	North America
Worm et al. 2009 (32)	Yes	1.80*	1.43	2.26	OR	No	Cohort	Europe, Australia, US	Europe & America
Duration of protease inhibitor use									
Engel et al., 2021 (43)	≥1 yr	2.4*	1.53	3.63	OR	No	Case-control	Switzerland	Europe
Lai et al., 2008 (36)	≥6 months	2.51	0.95	6.69	OR	No	Cohort	USA	North America
Worm et al., 2009 (32)	Per 3 months increase	219.30*	79.79	762.57	OR	No	Cohort	Europe, Australia, US	North America & Europe
Bucher et al., 2012 (45)	Per 3 months increase	1.02	0.93	1.12	OR	No	Case-control	Switzerland	Europe
Viral load									
Berquist et al., 2017 (38)	–	3.18*	1.12	9.08	OR	No	Case-control	Australia	Australia
Bucher et al. 2012 (45)	–	1.44*	1.27	1.89	OR	No	Case-control	Switzerland	Europe
Lai et al. 2008 (36)	≥400 copies/ml	1.40	0.38	5.35	OR	No	Cohort	USA	North America
Worm et al., 2009 (32)	continuous	0.18*	0.14	0.22	OR	No	Cohort	Europe, Australia, US	Europe & North America
Egana-Gorrono et al., 2012 (46)	–	1.204	0.15	1.97	OR	No	Case-control	Barcelona	Europe
Escaut et al., 2003 (47)	Unsuppressed (>1000 copies)	0.77	0.27	2.21	OR	No	Cohort	France	Europe
Trøseid et al., 2024 (53)	Suppressed (<50 copies)	8.44	0.47	150.38	OR	No	Nested cross-sectional	Norway	Europe
Urina-Jassir et al., 2023 (52)	Unsuppressed VL	0.49*	0.28	0.86	OR	No	Cohort	Colombia	South America
Trevillyan et al. (2013) (41)	–	0.85	0.45	1.61	OR	No	Matched case-control	Australia	Australia
Trevillyan et al. (2017) (40)	–	3.05*	1.05	8.84	OR	No	Matched case-control	Australia	Australia
LDL									
Bucher et al. 2012 (45)	–	1.04	0.99	1.10	OR	No	Case-control	Switzerland	Europe
Escaut et al. 2003 (47)	–	5.84*	2.44	14.01	OR	No	Cohort	France	Europe
Lai et al. 2008 (36)	≥100 mg/dl	6.10*	1.69	25.2	OR	No	Cohort	USA	North America
Trevillyan et al. (2013) (41)	–	0.31*	0.15	0.645	OR	No	Matched case-control	Australia	Australia
Trevillyan et al. (2017) (40)	–	1.00	0.41	2.45	OR	No	Matched case-control	Australia	Australia
Total cholesterol									
Bucher et al. 2012 (45)	Per 10 mg/dl	1.08	1.00	1.17	OR	No	Case-control	Switzerland	Europe
Escaut et al. 2003 (47)		3.19*	1.33	7.64	OR	No	Cohort	France	Europe
Lai et al. 2008 (36)	≥160 mg/dl	4.60*	1.55	16.70	OR	No	Cohort	USA	North America
May et al. 2007 (44)	–	1.31*	1.22	1.40	HR	No	Systematic review	Switzerland	Europe
Trevillyan et al. 2017 (40)	–	1.22	0.50	2.99	OR	No	Matched case-control	Australia	Australia
Triglycerides									
Bucher et al. 2012 (45)	–	0.99	0.97	1.01	OR	No	Case-control	Switzerland	Europe
Escaut et al. 2003 (47)	–	4.56*	1.90	10.92	OR	No	Cohort	France	Europe
Lai et al. 2008 (36)	≥130 mg/dl	2.65*	1.01	7.11	OR	No	Cohort	USA	North America
May et al. 2007 (44)	–	1.00	0.85	1.18	HR	No	Systematic review	Switzerland	Europe
Trevillyan et al. (2013) (41)	–	2.67*	1.29	5.50	OR	No	Matched case-control	Australia	Australia
Trevillyan et al. (2017) (40)	–	2.50*	1.01	6.19	OR	No	Matched case-control	Australia	Australia
HDL									
Bucher et al. 2012 (45)	–	0.96*	0.93	0.99	OR	No	Case-control	Switzerland	Europe
Escaut et al. 2003 (47)	–	0.70*	0.29	1.66	OR	No	Cohort	France	Europe
Lai et al. 2008 (36)	≥50 mg/dl	0.68	0.25	1.76	OR	No	Cohort	USA	North America
May et al. 2007 (44)	–	0.46*	0.34	0.61	HR	No	Systematic review	Switzerland	Europe
Trevillyan et al. (2013) (41)	–	2.02	0.82	4.97	OR	No	Matched case-control	Australia	Australia
Family history of CVD									
Bucher et al. 2012 (45)	Yes	2.06*	1.01	4.19	OR	No	Case-control	Switzerland	Europe
Berquist et al. 2017 (38)	Yes	4.1*	1.39	12.13	OR	No	Case-control	Australia	Australia
Trevillyan et al. (2013) (41)	Yes	6.22*	3.07	12.6	OR	No	Matched case-control	Australia	Australia
Engel et al. 2021 (43)	Yes	1.4	0.8	2.0	OR	No	Case-control	Switzerland	Europe
Worm et al. 2009 (32)	Yes	4.07*	3.08	5.37	OR	No	Cohort	Europe, Australia, US	Europe & North America
Lai et al. 2008 (36)	Yes	1.77	0.60	4.89	OR	No	Cohort	USA	North America
Egana-Gorrono et al. 2012 (46)	Yes	6.91*	0.83	5.76	OR	Yes	Case-control	Barcelona	Europe
Dyslipidemia									
Chammartin et al. 2022 (42)	Yes (lagged 36 month)	2.29*	1.63	3.21	HR	Yes	Cohort	Switzerland	Europe
Engel et al. 2021 (43)	Yes	1.92 *	1.41	2.63	OR	Yes	Case-control	Switzerland	Europe
Lui et al. 2023 (50)	Yes	3.30*	1.41	7.77	OR	No	Cohort	Hong kong	Asia
Weight/BMI									
Urina-Jassir et al. 2023 (52)	≥30 kg/m	2.95*	1.69	5.10	OR	Yes	Cohort	Colombia	South America
Fuchs et al. 2013 (48)	≥25 kg	1.80*	1.20	2.60	RR	Yes	Nested cross-sectional	Brazil	South America
Kaplan et al. 2007 (33)	≥25 kg	1.70*	1.20	2.41	OR	Yes	Cohort	USA	North America
Lai et al. 2008 (36)	≥24 kg	0.74	0.27	1.92	OR	No	Cohort	USA	North America
Escaut et al. 2003 (47)	–	0.53	0.22	1.27	OR	No	Cohort	France	Europe
May et al. 2007 (44)	–	1.11	0.98	1.25	HR	No	Systematic review	Switzerland	Europe
Trøseid et al. 2024 (53)	–	1.00	0.57	1.76	OR	No	Nested cross-sectional	Norway	Europe
Berquist et al., 2017 (38)	–	0.23*	0.092	0.59	OR	No	Case-control	Australia	Australia
History of AIDS									
Berquist et al., 2017 (38)	Yes	0.78	0.28	2.19	OR	Yes	Case-control	Australia	Australia
Egana-Gorrono et al. 2012 (46)	Yes	0.60	0.11	3.45	OR	Yes	Case-control	Barcelona	Europe
Kaplan et al. 2007 (33)	Yes	1.66*	1.22	2.27	OR	No	Cohort	USA	North America
Trøseid et al. 2024 (53)	Yes	256.60*	13.09	157	OR	No	Nested cross-sectional	Norway	Europe
Urina-Jassir et al. 2023 (52)	Yes	1.83*	1.07	3.12	OR	Yes	Cohort	Colombia	South America
Education									
Chammartin et al. 2022 (42)	(Vocational edu/high edu)	1.03	0.63	1.70	HR	Yes	Cohort	Switzerland	Europe
Kaplan et al. 2007 (33)	At least high school	1.08	0.77	1.52	OR	No	Cohort	USA	North America
Race									
Kaplan et al. 2007 (33)	(Non-white vs. white)	1.37	0.76	2.74	OR	No	Cohort	USA	North America
Fuchs et al. 2013 (48)	(Non-white vs. white)	1.1	0.8	1.4	RR	Yes	Nested cross-sectional	Brazil	South
Alcohol use									
Kaplan et al. 2007 (33)	Yes (Heavy use)	0.36*	0.13	0.95	OR	No	Cohort	USA	North America
Fuchs et al. 2013 (48)	Yes (Binge drinking)	0.6*	0.4	1.0	RR	Yes	Nested cross-sectional	Brazil	South America
Lai et al. 2008 (36)	Yes	0.65	0.16	3.91	OR	No	Cohort	USA	North America
Kaplan et al. 2007 (33)	Yes (Light to moderate use)	1.18	0.87	1.60	OR	No	Cohort	USA	North America
Hypertension									
Berquist et al., 2017 (38)	Yes	12.41*	3.37	45.65	OR	Yes	Case-control	Australia	Australia
Worm et al. 2009 (32)	Yes	30.85*	24.36	39.06	OR	No	Cohort	Europe, Australia, US	Europe, Australia, North America
Chammartin et al., 2022 (42)	Yes (lagged 36 months)	1.85*	1.33	2.57	HR	Yes	Cohort	Switzerland	Europe
Egana-Gorrono et al., 2012 (46)	Yes	3.58*	2.52	3.96	OR	Yes	Case-control	Barcelona	Europe
Engel et al., 2021 (43)	Yes	1.2	0.8	1.8	OR	No	Case-control	Switzerland	Europe
Fuchs et al., 2013 (48)	Yes	4.6*	2.7	7.9	RR	Yes	Nested cross-sectional	Brazil	South America
Lui et al., 2023 (50)	Yes	2.56*	1.09	6.04	OR	No	Cohort	Hong Kong	Asia
Mushin et al., 2023 (39)	Yes	10.3*	5.25	20.20	OR	No	Case-control	Australia	Australia
Trøseid et al., 2024 (53)	Yes	2.96*	1.54	5.69	OR	No	Nested cross-sectional	Norway	Europe
Trevillyan et al. (2013) (41)	Yes	5.46*	2.38	12.74	OR	No	Matched case-control	Australia	Australia
Trevillyan et al. (2017) (40)	Yes	9.69*	2.84*	33.07	OR	No	Matched case-control	Australia	Australia
Antiplatelet use									
Worm et al. 2009 (32)	Yes	111.57*	86.40	144.06	OR	No	Cohort	Europe, Australia, US	Europe, Australia, North America
Trevillyan et al. (2013) (41)	Yes	9.43*	3.11	33.84	OR	No	Matched case-control	Australia	Australia
Trevillyan et al. (2017) (40)	Yes	32.14*	3.78	273.56	OR	No	Matched case-control	Australia	Australia
Diabetes									
Berquist et al., 2017 (38)	Yes	2.87	0.59	14.03	OR	No	Case-control	Australia	Australia
Bucher et al., 2012 (45)	Yes	1.84	0.82	4.14	OR	Yes	Case-control	Switzerland	Europe
Egana-Gorrono et al., 2012 (46)	Yes	2.44*	2.3	5.78	OR	Yes	Case-control	Barcelona	Europe
Fuchs et al., 2013 (48)	Yes	4.7*	3.4	6.5	RR	Yes	Nested cross-sectional	Brazil	South America
May et al., 2007 (44)	Yes	1.67	0.75	4.03	HR	No	Systematic review	Switzerland	Europe
Urina-Jassir et al., 2023 (52)	Yes	2.50*	1.25	4.97	OR	Yes	Cohort	Colombia	South America
Trevillyan et al. (2013) (41)	Yes	1.82	0.69	4.71	OR	No	Matched case-control	Australia	Australia
Trevillyan et al. (2017) (40)	Yes	2.87*	0.59	14.03	OR	No	Matched case-control	Australia	Australia
Cocaine use									
Fuchs et al., 2013 (48) (past or current)	Yes	0.60	0.30	1.20	RR	Yes	Nested cross-sectional	Brazil	South America
Lai et al., 2008 (36) (Duration of cocaine use)	Yes (≥15 yrs)	7.75*	2.26	31.20	OR	No	Cohort	USA	North America
Mode of transmission (Intravenous drug users)	Yes								
Worm et al., 2009 (32)	Yes	0.34*	0.22	0.54	OR	No	Cohort	Europe, Australia, US	Europe, Australia, North America
Kaplan et al., 2007 (33)	Yes	0.94	0.67	1.32	OR	Yes	Cohort	USA	North America
Lipid lowering drugs									
Trøseid et al., 2024 (53)	Yes	11.74*	4.14	33.26	OR	No	Nested cross-sectional	Norway	Europe
Worm et al., 2009 (32)	Yes	18.84*	14.81	23.96	OR	No	Cohort	Europe, Australia, US	Europe, Australia, North America
CD4									
Bucher et al., 2012 (45)	Per 35 decreases	1.33*	1.11	1.59	OR	Yes	Case-control	Switzerland	Europe
Berquist et al., 2017 (38)	Decrease	0.17	0.065	0.42	OR	No	Case-control	Australia	Australia
Egana-Gorrono et al., 2012 (46)	Per 105 decreases	1.22*	1.06	1.05	OR	Yes	Case-control	Barcelona	Europe
Engel et al., 2021 (43)	<50 cells/ul	1.50*	0.95	2.5	OR	No	Case-control	Switzerland	Europe
Escaut et al., 2003 (47)	Decrease by 164 cells/mm^3^	3.60*	1.50	8.62	OR	No	Cohort	France	Europe
Lai et al., 2008 (36)	≥350 cells/mm	1.25	0.36	4.35	OR	No	Cohort	USA	North America
Worm et al., 2009 (32)	Per 26 decrease	0.08*	0.13	0.58	OR	No	Cohort	Europe, Australia, US	Europe, Australia, North America
Trevillyan et al. (2013) (41)	Per 55 decrease	0.48	1	500.00	OR	Yes	Matched case-control	Australia	Australia
Trevillyan et al. (2017) (40)	Decrease in CD4 count by 75	0.99	0.48	1.50	OR	No	Matched case-control	Australia	Australia
Nadir CD4									
Trevillyan et al. (2013) (41) (nadir)	Decrease by 14	0.16	1.04	0.35	OR	No	Matched case-control	Australia	Australia
Trevillyan et al. (2017) (40) (nadir)	Decrease by 16	6.11	2.38	15.70	OR	No	Matched case-control	Australia	Australia
Berquist et al., 2017 (38) (nadir)	–	1.72	0.70	4.24	OR	No	Case-control	Australia	Australia
Trøseid et al., 2024 (53) (nadir)	<200 cells/μl	2.8758	1.5046	5.4968	OR	No	Nested cross-sectional	Norway	Europe
Age	–								
Longenecker et al., 2022 (49)	–	1.089	0.948	1.250	OR	No	Cohort	Uganda	Africa
Berquist et al., 2017 (38)	–	1.00	0.41	2.44	OR	No	Case-control	Australia	Australia
Engel et al., 2021 (43)	–	1.26	1.14	1.39	OR	Yes	Case-control	Switzerland	Europe
Escaut et al., 2003 (47)	–	1.656	0.693	3.958	OR	No	Cohort	France	Europe
Chammartin et al., 2022 (42)	≥50	6.97	4.40	11.05	HR	Yes	Cohort	Switzerland	Europe
Fuchs et al., 2013 (48)	≥50 yr	29.85	19.65	45.35	RR	Yes	Nested cross-sectional	Brazil	South America
Lai et al., 2008 (36)	≥50 yr	3.89	1.05	13.3	OR	No	Cohort	USA	North America
Lui et al., 2023 (50)	≥55 yr	7.39	2.28	24.01	HR	No	Cohort	Hong Kong	Asia
Trøseid et al., 2024 (53)	≥12.4 yr increase (older)	1,556.006	596.95	4,055.89	OR	No	Nested cross-sectional	Norway	Europe
Worm et al., 2009 (32)	Per 13 yrs increase	5,168.047	4,156.43	6,424.706	OR	No	Cohort	Europe, Australia, US	Europe, Australia, North America
Urina-Jassir et al., 2023 (52)	≥50	4.96	3.29	7.45	OR	Yes	Cohort	Colombia	South America
Sex									
Chammartin et al., 2022 (42)	Female	0.30	0.17	0.53	HR	Yes	Cohort	Switzerland	Europe
Berquist et al., 2017 (38)	Female	1.64	0.34	8.00	OR	No	Case-control	Australia	Australia
Fuchs et al., 2013 (48)	Female	3.0	2.1	4.2	RR	Yes	Nested cross-sectional	Brazil	South America
Kaplan et al., 2007 (33)	Female	0.42	0.27	0.65	OR	Yes	Cohort	USA	North America
Lai et al., 2008 (36) (male)	Female	0.218	0.040	0.781	OR	Yes	Cohort	USA	North America
Longenecker et al., 2022 (49) (Female)	Female	4.144	0.383	44.855	OR	No	Cohort	Uganda	Africa
Pullinger et al., 2010 (51)	Female	0.5556	0.2942	0.817	Cohen D	Yes	Nested cross-sectional	USA	North America
Trøseid et al., 2024 (53)	Female	2.2	0.78	6.22	OR	No	Nested cross-sectional	Norway	Europe
Trevillyan et al. (2017) (40)	Female	1.5	0.31	7.33	OR	No	Matched case-control	Australia	Australia
Sexual orientation									
Worm et al., 2009 (32) (transmission homosexual)	Homosexuals	1.9475	1.5426	2.4587	OR	No	Cohort	Europe, Australia, US	Europe, Australia, North America
Egana-Gorrono et al. 2012 (46)	Homosexual	0.82	0.23	2.90	OR	No	Cohort	Europe, Australia, US	Europe, Australia, North America
Trøseid et al., 2024 (53)	Homosexuals	1.64	0.80	3.35	OR	No	Nested cross-sectional	Norway	Europe
Smoking									
Berquist et al., 2017 (38)	Yes (Current smoker, ever smoked)	1.64, 0.9	0.34, 0.33	8.00, 2.46	OR	No	Case-control	Australia	Australia
Chammartin et al., 2022 (42)	Yes (lagged 36 months)	1.71	1.22	2.38	HR	Yes	Case-control	Switzerland	Europe
Engel et al., 2021 (43) (past)	Yes	1.5	1.95	2.2	OR	No	Case-control	Switzerland	Europe
Engel et al., 2021 (43) (current)	Yes	1.9	1.4	3.0	OR	No	Case-control	Switzerland	Europe
Fuchs et al., 2013 (48) (lifetime smoking)	Yes	2.20	1.6	3.0	RR	Yes	Nested cross-sectional	Brazil	South America
Lai et al., 2008 (36) (ever smoked)	Yes	1.92	0.42	18.1	OR	No	Cohort	USA	North America
Trøseid et al., 2024 (53) (current)	Yes	1.9291	0.9744	3.8192	OR	No	Nested cross-sectional	Norway	Europe
Trevillyan et al. (2017) (40) current	Yes	1.4211	0.5857	3.8333	OR	No	Matched case-control	Australia	Australia
Mushin et al., 2023 (39) (past)	Yes	1.23	0.65	2.31	OR	No	Case-control	Australia	Australia
Mushin et al., 2023 (39) (current)	Yes	2.31	1.32	4.04	OR	No	Case-control	Australia	Australia
Egana-Gorrono et al., 2012 (46)	Yes	2.044	0.563	7.424	OR	Yes	Case-control	Barcelona	Europe
Escaut et al., 2003 (47) (current)	Yes	1.58	0.57	4.42	OR	No	Cohort	France	Europe
May et al., 2007 (44) (past)	Yes	1.04	0.81	1.35	HR	No	Systematic review	Switzerland	Europe
May et al., 2007 (44) (current)	Yes	2.04	1.61	2.57	HR	No	Systematic review	Switzerland	Europe
May et al., 2007 (44)		1.45	1.06	1.98	HR	No	Systematic review	Switzerland	Europe
Worm et al., 2009 (32) (past smoker)	Yes	2.4085	1.8849	3.0776	OR	No	Cohort	Europe, Australia, US	Europe, Australia, North America
Worm et al., 2009 (32) (current)	Yes	1.1025	0.8692	1.3984	OR	No	Cohort	Europe, Australia, US	Europe, Australia, North America
Duration of HIV infection									
Trøseid et al., 2024 (53) (in yrs)	High yrs with HIV	3,861.35	1,369.480	10,885.399	OR	No	Nested cross-sectional	Norway	Europe
Berquist et al., 2017 (38)	–	1.00	0.41	2.45	OR	No	Case-control	Australia	Australia
Trevillyan et al. (2013) (41)	A 9 yr increase	1.9272	1.1323	3.28	OR	No	Matched case-control	Australia	Australia
Trevillyan et al. (2017) (40)	Increase by 2.8 yrs	8.074	3.092	21.079	OR	No	Matched case-control	Australia	Australia
Fuchs et al., 2013 (48)	≥8 yrs	0.8	0.4	1.4	RR	Yes	Nested cross-sectional	Brazil	South America

* Statistically significant at α = 0.05.

To assess the certainty of the synthesized findings, the Grading of Recommendations, Assessment, Development, and Evaluation (GRADE) system was applied (27–30). The quality of evidence was graded using the JBI risk-of-bias tool, considering inconsistency (variability in reported effect sizes), indirectness (applicability of study findings to real-world settings), imprecision (wide confidence intervals affecting reliability), and publication bias (selective reporting or overrepresentation of positive findings). Studies that met all the five criteria were classified as high-certainty evidence, whereas those with multiple concerns were downgraded to moderate, low, or very low certainty.

Narrative synthesis was conducted using a structured stepwise approach designed to uncover and evaluate the relationships between CAD and its associated risk factors in PLWH. This approach was chosen because of the heterogeneity in study methodologies, exposure definitions, and outcome measures across the included studies. Where quantitative synthesis was feasible, the effect sizes were standardized, and the overall associations were quantified.

A combination of descriptive statistics, data visualization, predictive modeling, and advanced statistical tests was applied to achieve the research objectives. Bar charts were used to illustrate the composition of the risk classes, which showed the dominance of first- and third-class factors. Risk estimates (HR, OR, and HR) were standardized to ORs for comparison using 1/OR for OR <1 to standardize the effect direction and the confidence intervals were adjusted as follows [1/OR^2^] (21–24, 31). The aggregated ORs and heterogeneity (*I*^2^) were estimated using a random-effects meta-analysis model. The critical risk threshold (CRT) was identified at the 75th percentile of the cumulative Rw. Meta-analyses were conducted using R version 4.4.3 and ChatGPT-Python (version 3.x) interface, with a significance level of *α* = 0.05. We examined the impacts of variability in the mode of analysis (adjusted vs. unadjusted), and effect size type (OR, HR, and RR) on the distribution of significance. Furthermore, we undertook epidemiological synthesis and computed geotemporal trends per risk, risk responsiveness, predictive consistency (Rw), CI, and PHP per factor. Details of the epidemiological synthesis are provided in Supplementary File 2.

## 3 Results

### 3.1 Study selection

A total of 17,074 articles were retrieved, and 210 duplicates were removed. The remaining 16,864 articles were screened by title and abstract; 16,817 ineligible articles were excluded. Full-text screening was performed in 47 studies, of which 22 met the inclusion criteria (Figure 1). Fifteen risk factors, reported in at least two studies, were included in the narrative synthesis. Eleven risk factors, reported in three or more studies, were eligible for meta-analysis.

### 3.2 Study characteristics

The 22 studies (11 cohort, seven case-control, and four nested cross-sectional studies) included 103,370 participants. The studies were conducted across six continents: Europe (*n* = 7), North America (*n* = 6), Australia (*n* = 4), South America (*n* = 2), Asia (*n* = 1), and Africa (*n* = 1). One study (32) included participants from Europe, North America, and Australia. Five studies (33–37) were conducted in the United States, four studies (38–41) were conducted in Australia, and three studies (42–44) were conducted in Switzerland (Table 1).

### 3.3 Risk of bias

Fourteen studies (64%) were rated as having a low risk of bias; eight (36%) as moderate. All the studies recruited comparable exposed and unexposed groups. However, variations in the measurement tools, follow-up duration, and reporting completeness were noted (Supplementary File 3).

### 3.4 Risk factor associations

#### 3.4.1 Age, education, and race/ethnicity

Eleven studies examined the association between age and CAD. Of these, eight studies reported significant associations between age and CAD. Five were cohort studies (32, 36, 42, 50, 52), one was a case–control study (43), and two were nested cross-sectional studies (48, 53). Two studies reported non-significant associations between education and CAD (41, 42). No significant associations were reported between race/ethnicity and CAD (33, 48) (Table 2).

#### 3.4.2 Sex and sexual orientation

Of nine studies, five reported that sex was significantly associated with CAD (33, 36, 42, 48, 51). Three studies examined sexual orientation and CAD; one (36) showed a significant association.

#### 3.4.3 Smoking, alcohol, intravenous drug use, and cocaine

Among 12 studies on smoking, three reported significant associations with CAD (42, 43, 48). Of the three studies on alcohol, no significant associations were reported (36, 48). Of the two cohorts examining intravenous drug use, one reported that drug use was associated with a higher risk of CAD (36). One of the two studies reported that cocaine use was associated with an increased risk of CAD (40).

#### 3.4.4 CD4 count and viral load

Ten studies examined CD4 count, and six (32, 41, 45, 46, 47, 53) reported significant associations with CAD. Of the ten studies examining viral load, five reported significant associations with CAD (32, 38, 40, 45, 52). In two cohorts (32, 52), a higher viral load was reported to be protective against CAD (Table 2).

#### 3.4.5 Duration of HIV and history of AIDS-defining illness

Among the five studies on HIV duration and CAD, three (40, 41, 53) reported significant associations. Of five the studies on AIDS-defining illnesses, three (33, 52, 53) reported significant associations (Table 2).

#### 3.4.6 BMI, hypertension, and diabetes

Of eight studies on BMI, four reported significant associations with CAD (33, 38, 48, 52). Eleven studies examined the association between hypertension and CAD, but only one study did not find a significant association (43). Eight studies examined diabetes, of which three reported associations with CAD (46, 48, 52) (Table 2).

#### 3.4.7 Dyslipidemia and lipid profile

Three studies (42, 43, 50) reported that dyslipidemia was significantly associated with CAD. Of the five studies on LDL, three (36, 41, 47) reported significant associations. Five studies examined associations with total cholesterol, and four reported significant associations (36, 44, 45, 47). Four studies reported significant associations between triglycerides and CAD (36, 40, 41, 47). Among the five studies on HDL, two (44, 45) reported significant associations (Table 2).

#### 3.4.8 Family history of CVD

Among the seven studies investigating family history, four (32, 38, 41, 45) reported significant associations with CAD (Table 2).

#### 3.4.9 Use of antiplatelet and lipid-lowering medications

Three studies (32, 40, 41) reported significant associations between antiplatelet use and CAD. Two studies (32, 53) reported that the use of lipid-lowering drugs was associated with an increased risk of CAD (Table 2).

#### 3.4.10 Exposure to ART

Of the nine studies on protease inhibitors, four (32, 36, 38, 42) reported significant associations. Seven studies investigated the use of abacavir, of which five (38, 39, 42, 43, 45) reported significance. One of the two studies on NRTIs (36) and both studies on NNRTIs (32, 36) reported significant associations. Two of four studies on the duration of protease inhibitor use (32, 43) reported a significant association with increased risk of CAD (Table 2).

### 3.5 Meta-analysis of factors associated with CAD in PLWH

Sixteen factors met the eligibility criteria for the meta-analysis. Eleven factors [hypertension, advanced age (≥50 years), family history of CVD, dyslipidemia, diabetes, history of AIDS-defining illness, smoking, overweight/obesity, viral load, sexual orientation, and use of ART] were significantly associated with an increased risk of CAD (*p* < 0.05). Variations in the type of effect size (OR, HR, and RR) did not significantly affect the distribution of significance (*p* > 0.05). Likewise, for each of the factors, variability in the mode of analysis (adjusted vs. non-adjusted) did not impact the distribution of significance (*p* > 0.05). Among the significant factors, ORs ranged from 1.42 (95% CI: 1.06–8.85) for viral load to 4.96 (95% CI: 3.30–7.47) for advanced age. Of the 16 factors, meta-syntheses were associated with substantial heterogeneity (*I*^2^ ≥ 50%) (Table 3).

**Table 3:** Meta-analysis of potential risk factors of coronary artery disease (CAD) in people living with HIV (PLWH).

Factors	Reference	Odds ratio	95% CI	*I*^2^ (%)	*p*-value	Variance in effect size type (*p*-value)	Risk responsiveness (Ri)	Risk weight (Rw)	Crw	Geo- coverage	Temporalcoverage	Geo-temporal trend (GTT)
Age (years)	≥50	4.96	3.30–7.47	99.7	<0.001	0.461	0.73	3.58^U^	3.58	6/6	3/3	1.00 (Adj: 0.87)
Sex	Female	0.90	0.45–1.79	87.19	<0.001	0.186	NS	–	–	5/6	3/3	0.83
Sexual orientation	Homosexuals	1.87	1.50–2.32	0.00	0.392	1.000	0.33	0.62^l^	4.20	3/6	2/3	0.33 (Adj: 0.33)
Smoking	Currently/ever smoked	1.59	1.35–1.87	70.34	<0.001	0.260	0.53	0.84^L^	5.04	4/6	3/3	0.67 (Adj: 0.59)
Alcohol use	Yes	0.71	0.41–1.25	67.52	0.024	1.000	NS	–	–	2/6	2/3	0.22
Duration of HIV	Living longer with HIV	8.51	0.39–184.73	98.78	<0.001	0.819	NS	–	–	3/6	2/3	0.33
History of AID-defining illness	Yes	1.61	1.24–2.08	0.00	0.097	1.000	0.60	0.97**^L^**	6.01	4/6	3/3	0.67 (Adj: 0.64)
CD4 count	Lower values	0.78	0.35–1.75	96.83	<0.001	1.000	NS	–	–	3/6	3/3	0.5
Nadir CD4 count	Lower values	1.68	0.42–6.67	88.19	<0.001	1.000	NS	–	–	2/6	2/3	0.22
Viral load	High values	1.42	1.06–8.85	15.94%	0.212	1.000	0.38	0.53^l^	6.54	4/6	3/3	0.67 (Adj: 0.60)
Overweight/obesity	≥24 Kg/m^2^	1.81	1.33–2.48	38.67	0.097	>0.05	0.75	1.36**^M^**	7.90	2/6	3/3	0.33 (Adj: 0.53)
Hypertension	Yes	4.90	2.71–8.86	94.54	<0.001	0.885	0.91	4.50**^U^**	12.4	5/6	3/3	0.83 (Adj: 0.87)
Diabetes	Yes	2.64	1.86–3.74	45.73	0.082	0.368	0.50	1.32**^M^**	13.72	3/6	3/3	0.50 (Adj: 0.5)
Dyslipidemia	Yes	2.15	1.41–7.75	0.00	0.448	1.000	1.00	2.15**^U^**	15.87	3/6	2/3	0.33 (Adj: 0.67)
Family history of CVD	Yes	3.15	1.95–5.08	74.25	<0.001	1.000	0.71	2.24**^U^**	18.11	3/6	3/3	0.50 (Adj: 0.61)
ART exposure	Yes	1.68	1.43–1.97	58.81	<0.01	1.000	0.63	1.06**^l^**	19.17	2/6	3/3	0.33 (Adj: 0.48)
PI exposure	Yes	1.60	1.26–2.02	35.99	0.029	1.000	0.56	0.90^l^	–	4/6	3/3	0.67 (Adj:0.62)
Duration of PI exposure	High values	5.82	0.56–60.37	98.98	<0.001	1.000	NS	-	–	2/6	3/3	0.33
Abacavir exposure	Yes	1.60	1.21–2.11	64.38	0.004	1.000	0.71	1.14^l^	–	2/6	2/3	0.22 (Adj: 0.47)
CRT (clinical): 14.8												
CRT (community): 8.0												

### 3.6 Epidemiologic synthesis

#### 3.6.1 Risk responsiveness and geotemporal trend

Hypertension (Ri = 0.91) and dyslipidemia (Ri = 1.00) showed the highest risk responsiveness. Age (Ri = 0.73), overweight/obesity (Ri = 0.75), and family history of CVD (Ri = 0.71) showed good risk responsiveness.

Five factors (age, hypertension, smoking, history of AIDS-defining illness, and viral load) had a broad geographical coverage (4/6 continents). Sexual orientation, obesity, diabetes, and dyslipidemia showed an average coverage (3/6 continents). Nine risk factors were consistently reported across all three periods (2000–2010, 2011–2020, and ≥2021).

Age (GTT = 1.00) and hypertension (GTT = 0.83) showed the strongest geotemporal trends. Smoking (GTT = 0.67), history of AIDS-defining illness (GTT = 0.67), viral load (GTT = 0.67), diabetes (GTT = 0.5), and a family history of CVD (GTT = 0.5) showed average or above average geotemporal coverage (Table 3).

**Table 4:** The causality framework for deducing causality from the exposure-outcome association.

Risk factor	Strength of association (value & rating)	Temporality (value & rating)	Consistency value (rating)	Irreversibility among significant findings (value & rating)	Causality index	Risk class	Nature of risk factor	Public health priority
Advanced age (≥50)	4.96 (3)	0.64 (2)	0.73 (1)	Yes (1)	7/10	First class	1	8/15^l^
Sexual orientation (Homosexual)	1.87 (1)	0.67 (2)	0.33 (0)	Yes (1)	4/10	Third class	1	5/15^l^
Smoking	1.59 (1)	0.23 (1)	0.53 (0)	Yes (1)	3/10	Third class	5	8/18^TM^
History of AID-defining illness	1.61 (1)	0.4 (1)	0.60 (1)	Yes (1)	4/10	Third class	1	5/15^l^
Viral load	1.4 (1)	0.25 (1)	0.38 (0)	No (0)	2/10	Third class	5	7/15^l^
Overweight/obesity	1.8 (1)	0.75 (3)	0.75 (2)	Yes (1)	7/10	First class	5	12/15^T^
Hypertension	4.90 (3)	0.27 (1)	0.91 (2)	Yes (1)	7/10	First class	5	12/15^T^
Diabetes	2.64 (2)	0.13 (1)	0.50 (0)	Yes (1)	4/10	Third class	5	9/15^M^
Dyslipidemia	2.15 (2)	0.67 (2)	1.00 (3)	Yes (1)	8/10	First class	5	13/15^T^
Total cholesterol	1.56 (1)	0.40 (1)	0.80 (2)	Yes (1)	5/10	Second class	5	10/15^T^
Triglycerides	1.84 (1)	0.33 (1)	0.67 (1)	Yes (1)	4/10	Third class	5	10/15^T^
Family history of CVD	3.15 (2)	0.29 (1)	0.71 (1)	Yes (1)	5/10	Second class	1	6/15^l^
ART exposure	1.68 (1)	0.45 (1)	0.63 (1)	Yes (1)	4/10	Third class	1	5/15^l^
Abacavir exposure	1.60 (1)	0.14 (1)	0.71 (1)	Yes (1)	4/10	Third class	1	5/15

#### 3.6.2 Risk factor stratification, critical risk threshold and modelling CAD in PLWH

The Rw was used to classify risk factors. Hypertension (Rw = 4.5), age (Rw = 3.58), family history of CVD (Rw = 2.24), and dyslipidemia (Rw = 2.15) belonged to the high-risk group. Diabetes (Rw = 1.32) and obesity (Rw = 1.36) belonged to the middle risk group, whereas the rest belonged to the lower-risk group (Table 4, Supplementary File 2–Figure S1).

The critical risk threshold was 14.8 (75th percentile of cumulative Rw). Two “necessary causes” of CAD in PLWH emerged: advanced age (Ri = 0.73; GTT = 0.87) and hypertension (Ri = 0.91, GTT = 0.87). Component causes included any of the remaining factors, prioritized by Rw, GTT, and intervention feasibility. We assumed that HIV-related CAD is imminent in individuals whose risk factor combination equals the CRT. In addition, factors with a minimum GTT of 0.5 were preferred to improve the global utility of the model. A GTT of 0.5 signifies fair geographical (3/6 continents) and temporal coverage (2/3 periods). Clinically, the most parsimonic prediction model for HIV-related CAD includes the following factors: age, hypertension, dyslipidemia, family history of CVD, diabetes, and overweight/obesity. The primary prevention model included hypertension, dyslipidemia, and overweight/obesity (Supplementary File 2).

#### 3.6.3 Necessary vs. synergistic “causes”

In determining the path/model for HIV-related CAD, we asked the question “in how many ways can these eleven factors combine to produce the critical risk threshold (Rw ≥ 14.8)?” Following the principle of permutation and combination, there are over 130 ways in which these eleven risk factors could interact to produce an Rw of 14.8 (Supplementary File 2). There are over 130 ways in which these 11 factors can combine to meet the CRT, and in all these combinations, the two irreplaceable factors were age and hypertension.

#### 3.6.4 Causality index and public health priority

We further classified the 11 risk factors using CI and PHP. Similar to Rw, first-class factors included dyslipidemia, hypertension, advanced age, and obesity. In terms of PHP, hypertension, dyslipidemia, and overweight/obesity were critical contributors to HIV-related CAD, followed by diabetes, smoking, and viral load.

A mass prevention threshold of 8.0 was set, integrating both Rw and PHP to identify actionable causal paths for primary prevention. Full model details are provided in Supplementary File 2.

### 3.7 Certainty of evidence

The certainty of evidence was moderate to high for 10 of the 11 significant variables, suggesting that further research is unlikely to alter the conclusions. Evidence supporting viral load as a risk factor for HIV-related CAD was rated low. Three factors were upgraded owing to large effect sizes. Heterogeneity and imprecision were the primary reasons for downgrading (Table 5).

**Table 5:** Certainty of evidence for each risk factor underscoring the review findings.

Domains Factors	Limitation	Indirectness	Imprecision	Inconsistency	Publication bias	Strength of association (OR)	Certainty of evidence
Advanced age (≥50)	Not serious	Not serious	Not serious	serious	Unlikely	Large	High ↓ 1 ↑ 1
Sexual orientation (Homosexual)	Not serious	Not serious	Not serious	Not serious	Likely	Moderate	Moderate ↓1
Smoking	Not serious	Not serious	Not serious	Serious	Unlikely	Moderate	Moderate ↓1
History of AIDS-defining illness	Not serious	Not serious	Serious	Not serious	Unlikely	Moderate	Moderate ↓1
Viral load	Not serious	Not serious	Serious	Not serious	Unlikely	Small	Moderate ↓1
Body mass index	Not serious	Not serious	Serious	Not serious	Unlikely	Moderate	Moderate ↓1
Hypertension	Not serious	Not serious	Serious	Serious	Unlikely	Large	Moderate ↓2↑1
Diabetes	Not serious	Not serious	Serious	Not serious	Unlikely	Moderate	Moderate ↓1
Dyslipidemia	Not serious	Not serious	Serious	Not serious	Unlikely	Moderate	Moderate ↓1
Family history of cardiovascular disease	Not serious	Not serious	Serious	Serious	Unlikely	Large	Moderate ↓2↑1
Antiretroviral exposure	Not serious	Serious	Not serious	Serious	Unlikely	Moderate	Low ↓ 2

### 3.8 Comparison of classification systems

**Figure 2:**
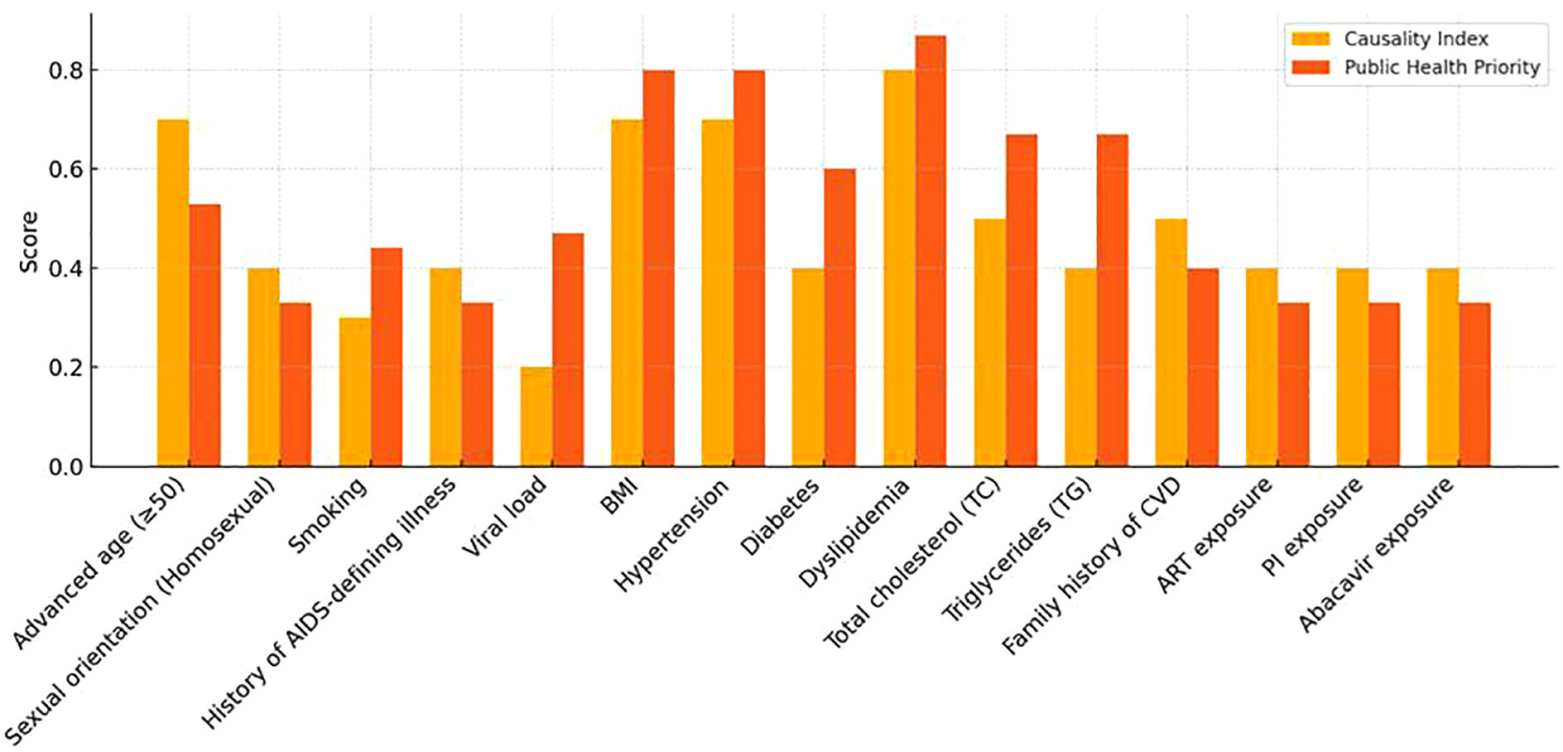
Comparison of the causality index score and public health priority score.

Two scoring systems were compared: an empirical model (based on effect size and meta-analysis results, maximum score = 10) and an integrated model, that included PHP. The goal was to classify the risk factors into relevant categories (Figure 2).

i. High CAD risk: hypertension (CI/PHP), age (CI), dyslipidemia (CI/PHP), obesity (CI/PHP), and viral load (PHP)
ii. Moderate CAD risk: diabetes (PHP), smoking (PHP), and family history (CI)
iii. Low CAD risk: remaining factors

## 4 Discussion

This systematic review and meta-analysis synthesized evidence on the risk factors for CAD among PLWH, integrating epidemiological models to enhance risk classification and prediction. The findings identified both traditional and HIV-specific factors with varying levels of predictive strength, causal relevance, and PHP. Advanced age and hypertension emerged as consistent and necessary causes, whereas dyslipidemia, diabetes, obesity, smoking, and viral load contributed as component or synergistic factors. By applying structured metrics—such as CI, risk responsiveness, and geotemporal coverage—this study offers a refined framework for understanding CAD risk in PLWH, with implications for clinical decision-making, public health policy, and future research.

### 4.1 Advanced age and hypertension

Advanced age and hypertension are well–known risk factors for CAD among PLWH, with high CI values and geotemporal coverage. While previous models included these factors in CVD prediction among PLWH (58), this is the first review to isolate their specific association with CAD in this population.

Contrary to prior hypotheses suggesting a higher CAD risk at younger ages (<55 years) (55), our findings affirm that advanced age (≥50 years) significantly increases CAD risk in PLWH. The biological mechanisms, vascular aging, endothelial dysfunction, and arterial stiffness, are well documented (56). Similarly, the pathophysiological role of hypertension in atherosclerosis and its universal inclusion in CVD risk models underscores its importance (54).

Both age and hypertension received high weights in prior models (54) owing to strong geotemporal coverage, predictive consistency, and low cost of assessment. The certainty of evidence for both is strong, indicating that future research is unlikely to change their classification. Althoug age is critical for risk prediction, its non-modifiable nature limits its relevance in primary prevention.

### 4.2 Family history of CVD, dyslipidemia, and diabetes mellitus

A family history of CVD, dyslipidemia, and diabetes also emerged as first-class CAD risk factors. PLWH with a family history of CVD had a threefold increased risk of developing CAD, consistent with general population studies (57–60). Bachmann et al. reported a 50% increase in lifetime mortality among patients with a history of premature CVD (55).

Dyslipidemia in PLWH was associated with a twofold increased risk of CAD, aligning with previous findings in ART-treated patients (61–63). Diabetes showed a threefold increase in CAD risk, corroborating the findings of Gupta et al (64). and supporting type 2 diabetes as a CAD risk equivalent (65). Mechanistically, chronic hyperglycemia, insulin resistance, and oxidative stress drive endothelial dysfunction and atherosclerosis (66).

Despite its predictive value, family history is non-modifiable and often excluded from clinical tools (50, 67–69). Dyslipidemia and diabetes are included more consistently; however, the moderate-to-high cost of dyslipidemia testing limits its use in LMICs. Our analysis suggests that, in the absence of age and hypertension, dyslipidemia or diabetes alone may be insufficient to indicate CAD risk in PLWH.

### 4.3 Obesity

Obesity ranked fifth the among risk factors, with obese PLWH experiencing approximately double the risk of CAD. This finding mirrors the general-population meta-analysis of 21 cohorts (70). Obesity contributes to CAD through increased blood volume, cardiac output, heart rate, and sodium retention, all promoting hypertension (71). Visceral and pericardial fat deposition correlates with plaque formation and vulnerability (72), whereas elevated fibrinogen, PAI-1, and coagulation factors increase thrombotic risk (73). The multifactorial effects of obesity reinforce hypertension as a “necessary cause” of HIV-related CAD. The moderate certainty of this evidence suggests that future findings may refine, but not reverse this association.

### 4.4 Smoking

Current or past smoking doubled HIV-related CAD risk, consistent with previous studies (74, 75). Smoking promotes vascular injury via oxidative stress, cytokine release, and endothelial dysfunction (76–78). Carbon monoxide and renal dysfunction further increase cardiovascular strain (79, 80). Smoking is relatively inexpensive to assess, supporting its inclusion in most CAD prediction models (54). The moderate certainty of evidence implies potential refinement, but not invalidation, of smoking risk status.

### 4.5 Viral load

Viral load has emerged as a third-class CAD risk factor, a novel finding for PLWH. Viral load has a low predictive value, explaining its omission from key models (e.g., D:A:D, SMART) (54). Mechanistically, viral proteins (Tat, gp120) trigger monocyte activation and endothelial apoptosis (81, 82). Early ART and sustained suppression mitigate these effects (83, 84). A high viral load likely acts as a potentiating factor, and is not a direct cause. Given its central role in HIV management, viral load qualifies as a PHP, despite a lower PHP score. The moderate certainty of evidence suggests that this association may evolve with further research.

### 4.6 History of AIDS-defining illness

A history of AIDS-defining illness doubled the CAD risk, consistent with the findings of the MACS cohort study (33). Chronic inflammation, activated T cells, and upregulated procoagulants drive atherosclerosis and thrombosis (85–87). Despite its biological plausibility, its non-modifiable nature and low predictive weight limit its value as a primary prevention target.

### 4.7 ART exposure

ART use was associated with a 1.7-fold increased in CAD risk, albeit inconsistently, —aligning with earlier data on subclinical atherosclerosis (88). Protease inhibitors disrupt lipid metabolism, impair glucose uptake, and induce mitochondrial toxicity (89–92). As a third-class factor, ART exposure gains predictive value only when combined with first-class risk factors, explaining its exclusion from some existing models (50, 54).

### 4.8 Sexual orientation (homosexual)

Homosexual orientation was associated with a twofold increase in CAD risk among PLWH, reflecting broader disparities in sexual minority populations (93). Though not universally applicable, regional prevalence and behavioral profiles may enhance its contextual relevance (94–97). This association is unlikely to be overturned, although its Rw may vary by setting.

#### 4.8.1 Antiplatelet and lipid lowering medications

One of the most important, although, accidental finding in this review is the fact that lipid lowering and antiplatelet medication constituted protective factors. Individuals on antiplatelet and lipid lowering medications were 36— and 18 —times, respectively, less likely to have CAD compare to their counterparts on no such medications. The efficacy of antiplatelet medication for secondary prevention of CAD is well-established (98). Low-dose aspirin is recommended for secondary prevention in patients with-established CAD, including those with a history of a heart attack, stroke, or angina. It works by inhibiting platelet aggregation, which helps prevent blood clots from forming in narrowed arteries. This can reduce the risk of future heart attack, stroke, and other cardiovascular events. Aspirin is often used in combination with other antiplatelet medications such as clopidogrel (98). Although effective, long-term aspirin use can increase the risk of bleeding, particularly in the stomach and intestines. Although its use in secondary prevention of CAD is justifiable, its benefits for primary prevention are a subject of debate. Similarly, the use of lipid-lowering medications (e.g., statins) for individuals at risk of CAD or established CAD is common practice (99). The goal of this practice is to significantly lower low-density lipoprotein cholesterol levels, thereby increasing the risk or progression of CAD (100). Therefore, there is a need to account for the role of protective factors, such as the use of antiplatelet and lipid-lowering medications, when modelling HV-related CAD, as this will enhance model performance by reducing the false positives.

### 4.9 Clinical relevance

This review confirms that Rw and CI are useful for stratifying CAD risk among PLWH (101). A CI ≥ 7 indicates high PHP risk factors that require targeted screening. PLWH older than 50 years with hypertension constitute a core surveillance group. This is especially true in the presence of additional factors such as a family history of CVD and/or dyslipidemia. Emphasizing modifiable behaviors risk factors such as hypertension, dyslipidemia, diabetes, obesity, smoking, and viral load aligns with existing prevention frameworks (102, 103).

### 4.10 Public health implications

A PHP threshold ≥8 supports community level interventions targeting high-CI modifiable risks (104, 105). Mass media, educational initiatives, and counseling have demonstrated efficacy in promoting behavioral change, particularly in resource-limited settings. In low- and middle-income countries (LMICs), where health systems frequently encounter infrastructural and workforce challenges, cost-effective, population-based strategies are increasingly vital. For instance, mass media campaigns can enhance awareness of cardiovascular risk factors, while programs based in schools and workplaces can encourage healthy lifestyles and facilitate early detection. Surveillance should monitor established and emerging risks such as alcohol use and ART-induced metabolic syndromes (106). Incorporating CAD risk surveillance into existing primary healthcare systems and national health surveys in LMICs could improve early detection and prevention on a large scale. Enhancing data systems and training community health workers to screen for risk factors can mitigate access to care disparities. Ultimately, translating these findings into national guidelines and policies could guide targeted resource allocation and reduce the long-term burden of coronary artery disease among vulnerable populations.

### 4.11 Policy implications

A CI: ≥7 threshold offers policymakers a quantitative tool for resource allocation and guideline development. Emphasizing behavioral determinants in planning, such as smoking cessation and viral-load monitoring, can curb CAD morbidity and mortality in HIV populations (107).

### 4.12 Recommendations

Based on the analyses conducted in this study, the following recommendations are proposed to enhance the understanding, prediction, and management of CAD risk in HIV-infected individuals living with HIV.

1. Validation of the epidemiologic metrics
  Geotemporal coverage, causality index, risk responsiveness, and public health priority. Although the new metrics provide deeper insights for assessing exposure-outcome association, there is a need to ascertain their definitive cut-off and compare them with the current practice of the sole use of statistical significance in ranking their importance and in the construction of predictive models for diseases.
2. Development of clinical decision tools
  Upon validation, the optimum critical risk threshold can serve as a critical cutoff for distinguishing individual at various risk levels. This knowledge can be used to develop simple clinical tools to assist healthcare professionals in risk stratification. Interactive calculators or dashboards can aid in identifying patients at higher risk and prioritizing preventive interventions accordingly.
3. Expanding the dataset for greater statistical power
  The dataset analyzed in this study contained only 11 risk factors, with 3–11 studies, which may limit the performance of the model and the generalizability of the findings. Future research should focus on collecting larger and more diverse datasets (using the epidemiological triangle or biopsychosocial model to inform variable sampling) to ensure that the identified relationships are consistent across a broader population. Expanding the dataset would provide more reliable estimates of the influence of the causality index on CAD risk and improve the ability of the model to discriminate between various level of CAD risk.
4. Incorporating additional predictors
  The current analysis relies primarily on the causality index and the risk class. Additional predictors, such as inflammatory biomarkers, treatment adherence data, alcohol use, substance use, air pollution and social determinants, may improve model performance. Such variables could provide a more nuanced understanding of risk dynamics and improve classification accuracy.
5. Standard definition of CAD and use of epidemiological model
  Future primary studies should employ standard diagnostic criteria for CAD to ensure a good degree of consistency in future syntheses. Owing to the irregular sampling of behavioural and socioeconomic determinants, future research examining factors associated with HIV-related CAD should employ relevant theoretical models such as the epidemiological triangle or biopsychosocial model to ensure exhaustive sampling of exposure variables, including social and environmental determinant. As a rule of thumb, an exposure-outcome (non-communicable disease) association should sample at least one each of biological, behavioural, social and environmental variables.
6. Implementation of ongoing monitoring and validation
  CAD risk profiles may evolve as HIV treatment improves and metabolic complication rates change. Ongoing data collection, monitoring, and model revalidation are essential to ensure that predictions remain accurate and actionable over time.
7. Conduct population-specific sub-analyses
  HIV is a heterogeneous condition that affects individuals differently depending on their age, sex, treatment history, and comorbidities. Conducting sub-analyses within specific demographic and clinical subgroups may uncover differential CAD risk patterns and inform more personalized and effective intervention strategies.
8. Accounting for protective factors in construction of global prediction model for CAD
  Failure to account for protective factors when constructing a global prediction model for HIV-related CAD could affect model performance by increasing the number of false positives. Therefore, a methodological innovation is required to include these factors in clinical prediction models for CAD in PLWH.

Addressing the model's limitations and expanding the data sources could significantly improve predictive performance and contribute to more effective CAD prevention efforts in this vulnerable population.

### 4.13 Limitations

Despite its methodological rigor, this study has some limitations. Internal metrics Rw, CI, and PHP require external validation. Inconsistent definitions of CAD may have affected the outcomes of the studies included in this review. Moreover, the strict criteria were impractical because of the limited number of eligible studies. Most factors lack representation from multiple continents, thus impacting their global applicability. Subjectivity in assessing the critical risk thresholds may affect the robustness of the model. However, a modest effort was made to account for this disparity by computing the geotemporal trends. Nevertheless, the validated framework may be applied differently to LMICs. In addition, the inability to account for protective factors such as the use of lipid-lowering and antiplatelet medication represents a limitation for which a methodological innovation is warranted. These findings should be validated in independent cohort studies.

## 5 Conclusion

This review identified advanced age and hypertension as primary risk factors for CAD in PLWH. Additional significant factors include dyslipidemia, obesity, diabetes, smoking, viral load, and ART exposure. The most parsimonic clinical prediction model included age, hypertension, dyslipidemia, family history of CVD, diabetes, and obesity as risk factors for HIV-related CAD. The most efficient models for primary prevention include hypertension, dyslipidemia, and obesity. These findings highlight the importance of risk stratification by integrating biological plausibility, PHP, and causality into risk assessments. The use of antiplatelet or lipid-lowering medications is protective against CAD in PLWH. Therefore, a methodological innovation that accounts for the use of antiplatelet and lipid-lowering medications in prediction models is essential to reduce false positives. Behavioral and socioeconomic factors were poorly sampled in eligible studies. Future primary studies should be guided by relevant epidemiological models to ensure exhaustive sampling of the exposure variables. Future studies should employ stricter assessment criteria to improve homogeneity and external validity.

## Data Availability

The original contributions presented in the study are included in the article/Supplementary Material, further inquiries can be directed to the corresponding author.

## References

[1] World Health Organization. Global Health Sector Strategies on, Respectively, HIV, Viral Hepatitis and Sexually Transmitted Infections for the Period 2022–2030. Geneva: World Health Organization (2022).

[2] American Health Association. As HIV patients live longer, heart disease might be their next challenge. (2019).

[3] Triant VA Lee H Hadigan C Grinspoon SK. Increased acute myocardial infarction rates and cardiovascular risk factors among patients with human immunodeficiency virus disease. J Clin Endocrinol Metab. (2007) 92(7):2506–12. 10.1210/jc.2006-2190 17456578 PMC2763385

[4] Friis-Møller N Thiebaut R Reiss P El-Sadr W Weber R D’Arminio Monforte A Predicting the risk of coronary heart disease (CHD) in HIV-infected patients: the D: a: d CHD risk equation. 14th Conference on Retroviruses and Opportunistic Infections (2007). Vol. 27.

[5] Shah AS Stelzle D Lee KK Beck EJ Alam S Clifford S Global burden of atherosclerotic cardiovascular disease in people living with HIV: systematic review and meta-analysis. Circulation. (2018) 138(11):1100–12. 10.1161/CIRCULATIONAHA.117.033369 29967196 PMC6221183

[6] Hsue PY Deeks SG Hunt PW. Immunologic basis of cardiovascular disease in HIV-infected adults. J Infect Dis. (2012) 205(Suppl 3):S375–82. 10.1093/infdis/jis200 22577211 PMC3349295

[7] Freiberg MS Chang CC Kuller LH Skanderson M Lowy E Kraemer KL HIV Infection and the risk of acute myocardial infarction. JAMA Intern Med. (2013) 173(8):614–22. 10.1001/jamainternmed.2013.3728 23459863 PMC4766798

[8] Bekker LG Alleyne G Baral S Cepeda J Daskalakis D Dowdy D Advancing global health and strengthening the HIV response in the era of the sustainable development goals: the international AIDS society-lancet commission. Lancet. (2018) 392(10144):312–58. 10.1016/S0140-6736(18)31070-5 30032975 PMC6323648

[9] Nou E Lo J Grinspoon SK. Inflammation, immune activation, and cardiovascular disease in HIV. AIDS. (2016) 30(10):1495–509. 10.1097/QAD.0000000000001109 27058351 PMC4889507

[10] Drain PK Dorward J Bender A Lillis L Marinucci F Sacks J Point-of-care HIV viral load testing: an essential tool for a sustainable global HIV/AIDS response. Clin Microbiol Rev. (2019) 32(3):e00097–18. 10.1128/CMR.00097-18 31092508 PMC6589862

[11] Mdodo R Frazier EL Dube SR Mattson CL Sutton MY Brooks JT Cigarette smoking prevalence among adults with HIV compared with the general adult population in the United States: cross-sectional surveys. Ann Intern Med. (2015) 162:335–44. 10.7326/M14-0954 25732274

[12] Feinstein MJ Bogorodskaya M Bloomfield GS Vedanthan R Siedner MJ Kwan GF Cardiovascular complications of HIV in endemic countries. Curr Cardiol Rep. (2016) 18(11):113. 10.1007/s11886-016-0794-x 27730474 PMC6717318

[13] Triant VA Lyass A Hurley LB Borowsky LH Ehrbar RQ He W Cardiovascular risk estimation is suboptimal in people with HIV. J Am Heart Assoc. (2024) 13(10):e029228. 10.1161/JAHA.123.029228 38761071 PMC11179796

[14] Vachiat A McCutcheon K Tsabedze N Zachariah D Manga P. HIV And ischemic heart disease. J Am Coll Cardiol. (2017) 69(1):73–82. 10.1016/j.jacc.2016.09.979 28057253

[15] Mills EJ Bärnighausen T Negin J. HIV And aging—preparing for the challenges ahead. N Engl J Med. (2012) 366(14):1270–3. 10.1056/NEJMp1113643 22475591

[16] Murray A Huang MJ Hardnett F Sutton MY. Strengthening HIV knowledge and awareness among undergraduate students at historically black colleges and universities. J Health Disparities Res Pract. (2014) 7(4):4. Available online at: https://oasis.library.unlv.edu/jhdrp/vol7/iss4/4

[17] Hill AB. The environment and disease: association or causation?. 10.1177/003591576505800503 PMC1898525 14283879

[18] Rothman KJ. Causes. Am J Epidemiol. (1976) 104(6):587–92. 10.1093/oxfordjournals.aje.a112335 998606

[19] Nweke M Ukwuoma M Adiuku-Brown AC Ugwu P Nseka E. Characterization and stratification of the correlates of postpartum depression in sub-Saharan Africa: a systematic review with meta-analysis. Womens Health (Lond). (2022) 18:17455057221118773. 10.1177/17455057221118773 36039898 PMC9434669

[20] Moher D Liberati A Tetzlaff J Altman DG, PRISMA Group. Preferred reporting items for systematic reviews and meta-analyses: the PRISMA statement. BMJ. (2009) 339:b2535. 10.1136/bmj.b2535 19622551 PMC2714657

[21] Nweke M van Vuuren M Bester K Maritz A van Vuuren L Vilakazi Y Association between socio-demographic and injury factors, and physical activity behaviour in people with spinal cord injury: a theory-informed systematic review and meta-analysis. BMC Sports Sci Med Rehabil. (2025) 17(1):179. 10.1186/s13102-024-01021-1 40611335 PMC12224392

[22] Nweke M Mshunqane N. Characterization and stratification of risk factors of stroke in people living with HIV: a theory-informed systematic review. BMC Cardiovasc Disord. (2025) 25(1):405. 10.1186/s12872-025-04833-2 40426038 PMC12107966

[23] Nweke M Oyirinnaya P Nwoha P Mitha SB Mshunqane N Govender N Development of a high-performing, cost-effective and inclusive afrocentric predictive model for stroke: a meta-analysis approach. BMC Neurol. (2025) 25(1):282. 10.1186/s12883-025-04229-x 40624481 PMC12232583

[24] Nwagha T Nweke M. Stratification of risk factors of lung cancer-associated venous thromboembolism and determining the critical point for preemptive intervention: a systematic review with meta-analysis. Clin Med Insights Oncol. (2023) 17:11795549231175221. 10.1177/11795549231175221 37426681 PMC10328178

[25] Moola S Munn Z Tufanaru C Aromataris E Sears K Sfetcu R Chapter 7. Systematic reviews of etiology and risk. In: Aromataris E Munn Z, editors. Joanna Briggs Institute Reviewer’s Manual. Adelaide: The Joanna Briggs institute (2017). p. 211–46. Available online at: https://reviewersmanual.joannabriggs.org/ (Accessed July 31, 2025).

[26] Chen H Cohen P Chen S. How big is a big odds ratio? Interpreting the magnitudes of odds ratios in epidemiological studies. Emerg Infect Dis. (2010) 16(6):e24. 10.1080/03610911003650383

[27] Guyatt GH Oxman AD Kunz R Woodcock J Brozek J Helfand M GRADE Guidelines: 7. Rating the quality of evidence—inconsistency. GRADE guidelines. J Clin Epidemiol. (2011) 64(12):1294–302. 10.1016/j.jclinepi.2011.03.017 21803546

[28] Guyatt GH Oxman AD Kunz R Woodcock J Brozek J Helfand M GRADE Guidelines: 8. Rating the quality of evidence—indirectness. GRADE guidelines. J Clin Epidemiol. (2011) 64(12):1303–10. 10.1016/j.jclinepi.2011.04.014 21802903

[29] Guyatt GH Oxman AD Kunz R Brozek J Alonso-Coello P Rind D RADE Guidelines 6. GRADE guidelines 6. GRADE guidelines 6. Rating the quality of evidence—imprecision. J Clin Epidemiol. (2011) 64(12):1283–93. 10.1016/j.jclinepi.2011.01.012 21839614

[30] Guyatt GH Oxman AD Montori V Vist G Kunz R Brozek J GRADE Guidelines: 5. Rating the quality of evidence—publication bias. J Clin Epidemiol. (2011) 64(12):1277–82. 10.1016/j.jclinepi.2011.01.011 21802904

[31] Ibeneme SC Odoh E Martins N Ibeneme GC. Developing an HIV-specific falls risk prediction model with a novel clinical index: a systematic review and meta-analysis method. BMC Infect Dis. (2024) 24(1):1402. 10.1186/s12879-024-10141-5 39696054 PMC11653889

[32] Worm SW De Wit S Weber R Sabin CA Reiss P El-Sadr W Diabetes mellitus, preexisting coronary heart disease, and the risk of subsequent coronary heart disease events in patients infected with human immunodeficiency virus: the Data Collection on Adverse Events of Anti-HIV Drugs (D:A:D) Study. Circulation. (2009) 119(6):805–11. 10.1161/CIRCULATIONAHA.108.790857 19188509 PMC2715841

[33] Kaplan RC Kingsley LA Sharrett AR Li X Lazar J Tien PC Ten-year predicted coronary heart disease risk in HIV-infected men and women. Clin Infect Dis. (2007) 45(8):1074–81. 10.1086/521935 17879928

[34] Dale SK Weber KM Cohen MH Brody LR. Abuse, nocturnal stress hormones, and coronary heart disease risk among women with HIV. AIDS Care. (2017) 29(5):598–602. 10.1080/09540121.2016.1241378 27733045 PMC5699459

[35] Freiberg MS Chang CC Skanderson M McGinnis K Kuller LH Kraemer KL The risk of incident coronary heart disease among veterans with and without HIV and hepatitis C. Circ Cardiovasc Qual Outcomes. (2011) 4(4):425–32. 10.1161/CIRCOUTCOMES.110.957415 21712519 PMC3159506

[36] Lai S Fishman EK Lai H Moore R Cofrancesco JJ Pannu H Long-term cocaine use and antiretroviral therapy are associated with silent coronary artery disease in African Americans with HIV infection who have no cardiovascular symptoms. Clin Infect Dis. (2008) 46(4):600–10. 10.1086/526782 19641630 PMC2716694

[37] Hadigan C Meigs JB Wilson PW D’Agostino RB Davis B Basgoz N Prediction of coronary heart disease risk in HIV-infected patients with fat redistribution. Clin Infect Dis. (2003) 36(7):909–16. 10.1086/368185 12652392

[38] Berquist VL Hearps AC Ford P Jaworowski A Leishman SJ Hoy JF Porphyromonas gingivalis antibody levels and diagnosis of coronary artery disease in HIV-positive individuals. J Periodont Res. (2017) 52(5):930–5. 10.1111/jre.12460 28397248

[39] Mushin AS Trevillyan JM Lee SJ Hearps AC Hoy JF. Factors associated with the development of coronary artery disease in people with HIV. Sex Health. (2023) 20(5):470–4. 10.1071/SH23043 37394729

[40] Trevillyan JM Gardiner EE Andrews RK Maisa A Hearps AC Al-Tamimi M Decreased levels of platelet-derived soluble glycoprotein VI detected prior to the first diagnosis of coronary artery disease in HIV-positive individuals. Platelets. (2017) 28(3):301–4. 10.1080/09537104.2016.1237627 27848272

[41] Trevillyan JM Cheng AC Hoy JF. Abacavir Exposure and cardiovascular risk factors in HIV-positive patients with coronary heart disease: a retrospective case–control study. Sex Health. (2013) 10(2):97–101. 10.1071/SH12081 23256968

[42] Chammartin F Darling K Abela IA Battegay M Furrer H Calmy A Swiss HIV cohort study. CD4:d8 ratio and CD8 cell count and their prognostic relevance for coronary heart disease events and stroke in antiretroviral-treated individuals: the Swiss HIV cohort study. J Acquir Immune Defic Syndr. (2022) 91(5):508–15. 10.1097/QAI.0000000000003094 36150371 PMC7613804

[43] Engel T Raffenberg M Schoepf IC Kootstra NA Reiss P Thorball CW Swiss HIV cohort study. Telomere length, traditional risk factors, factors related to human immunodeficiency virus (HIV) and coronary artery disease events in Swiss persons living with HIV. Clin Infect Dis. (2021) 73(7):e2070-6–e2076. 10.1093/cid/ciaa1034 32725240

[44] May M Sterne JA Shipley M Brunner E d’Agostino R Whincup P A coronary heart disease risk model for predicting the effect of potent antiretroviral therapy in HIV-1 infected men. Int J Epidemiol. (2007) 36(6):1309–18. 10.1093/ije/dym135 17652317

[45] Bucher HC Richter W Glass TR Magenta L Wang Q Cavassini M Small dense lipoproteins, apolipoprotein B, and risk of coronary events in HIV-infected patients on antiretroviral therapy: the Swiss HIV cohort study. J Acquir Immune Defic Syndr. (2012) 60(2):135–42. 10.1097/QAI.0b013e31824476e1 22156913

[46] Egaña-Gorroño L Martínez E Escribà T Calvo M Gatell JM Arnedo M. Association study of lipoprotein(a) genetic markers, traditional risk factors, and coronary heart disease in HIV-1-infected patients. Front Immunol. (2012) 3:367. 10.3389/fimmu.2012.00367 23230442 PMC3515864

[47] Escaut L Monsuez JJ Chironi G Merad M Teicher E Smadja D Coronary artery disease in HIV-infected patients. Intensive Care Med. (2003) 29(6):969–73. 10.1007/s00134-003-1740-0 12739013

[48] Fuchs SC Alencastro PR Ikeda ML Barcellos NT Wolff FH Brandão AB Risk of coronary heart disease among HIV-infected patients: a multicenter study in Brazil. Sci World J. (2013) 2013:163418. 10.1155/2013/163418 PMC3809373 24223499

[49] Longenecker CT Bogorodskaya M Margevicius S Nazzinda R Bittencourt MS Erem G Sex modifies the association between HIV and coronary artery disease among older adults in Uganda. J Int AIDS Soc. (2022) 25(1):e25868. 10.1002/jia2.25868 34995413 PMC8741262

[50] Lui G Leung HS Lee J Wong CK Li X Ho M An efficient approach to estimate the risk of coronary artery disease for people living with HIV using machine-learning-based retinal image analysis. PLoS One. (2023) 18(2):e0281701. 10.1371/journal.pone.0281701 36827291 PMC9955663

[51] Pullinger CR Aouizerat BE Gay C Coggins T Movsesyan I Davis H Metabolic abnormalities and coronary heart disease risk in human immunodeficiency virus–infected adults. Metab Syndr Relat Disord. (2010) 8(3):279–86. 10.1089/met.2009.0094 20235745 PMC3085320

[52] Urina-Jassir M Patiño-Aldana AF Herrera-Parra LJ Hernández Vargas JA Trujillo-Cáceres SJ Valbuena-García AM Factors associated with coronary artery disease among people living with human immunodeficiency virus: results from the Colombian HIV/AIDS registry. Int J Cardiol CardioVasc Risk Prev. (2023) 18:200205. 10.1016/j.ijcrp.2023.200205 37664166 PMC10469745

[53] Trøseid M Molinaro A Gelpi M Vestad B Kofoed KF Fuchs A Gut microbiota alterations and circulating imidazole propionate levels are associated with obstructive coronary artery disease in people with HIV. J Infect Dis. (2024) 229(3):898–907. 10.1093/infdis/jiad604 38195204 PMC10938217

[54] Yu J Liu X Zhu Z Yang Z He J Zhang L Prediction models for cardiovascular disease risk among people living with HIV: a systematic review and meta-analysis. Front Cardiovasc Med. (2023) 10:1138234. 10.3389/fcvm.2023.1138234 37034346 PMC10077152

[55] Bachmann JM Willis BL Ayers CR Khera A Berry JD. Association between family history and coronary heart disease death across long-term follow-up in men: the cooper center longitudinal study. Circulation. (2012) 125(25):3092–8. 10.1161/CIRCULATIONAHA.111.065490 22623718 PMC3631594

[56] Kemp CD Conte JV. The pathophysiology of heart failure. Cardiovasc Pathol. (2012) 21(5):365–71. 10.1016/j.carpath.2011.11.007 22227365

[57] Valerio L Peters RJ Zwinderman AH Pinto-Sietsma SJ. Association of family history with cardiovascular disease in hypertensive individuals in a multiethnic population. J Am Heart Assoc. (2016) 5(12):e004260. 10.1161/JAHA.116.004260 28003252 PMC5210427

[58] Lloyd-Jones DM Nam BH D’Agostino RB Sr Levy D Murabito JM Wang TJ Parental cardiovascular disease as a risk factor for cardiovascular disease in middle-aged adults: a prospective study of parents and offspring. JAMA. (2004) 291(18):2204–11. 10.1001/jama.291.18.2204 15138242

[59] Marenberg ME Risch N Berkman LF Floderus B de Faire U. Genetic susceptibility to death from coronary heart disease in a study of twins. N Engl J Med. (1994) 330(15):1041–6. 10.1056/NEJM199404143301503 8127331

[60] Andresdottir MB Sigurdsson G Sigvaldason H Gudnason V, Reykjavik Cohort Study. Fifteen percent of myocardial infarctions and coronary revascularizations explained by family history unrelated to conventional risk factors: the Reykjavik cohort study. Eur Heart J. (2002) 23(21):1655–63. 10.1053/euhj.2002.3235 12398822

[61] Di Lenarda F Balestrucci A Terzi R Lopes P Ciliberti G Marchetti D Coronary artery disease, family history, and screening perspectives: an up-to-date review. J Clin Med. (2024) 13(19):5833. 10.3390/jcm13195833 39407893 PMC11477357

[62] Grand M Bia D Diaz A. Cardiovascular risk assessment in people living with HIV: a systematic review and meta-analysis of real-life data. Curr HIV Res. (2020) 18(1):5–18. 10.2174/1570162X17666191212091618 31830884

[63] Dubé MP Stein JH Aberg JA Fichtenbaum CJ Gerber JG Tashima KT Guidelines for the evaluation and management of dyslipidemia in human immunodeficiency virus (HIV)-infected adults receiving antiretroviral therapy: recommendations of the HIV medical association of the infectious disease society of America and the adult AIDS clinical trials group. Clin Infect Dis. (2003) 37(5):613–27. 10.1086/378131 12942391

[64] Gupta N Elnour AA Sadeq A Gupta R. Diabetes and the heart: coronary artery disease. E-J Cardiol Pract. (2022) 22(10):112–20. Available online at: https://www.escardio.org/Journals/E-Journal-of-Cardiology-Practice/Volume-22/diabetes-and-the-heart-coronary-artery-disease#

[65] National Cholesterol Education Program (NCEP) Expert Panel on Detection, Evaluation, and Treatment of High Blood Cholesterol in Adults (Adult Treatment Panel III). Third report of the national cholesterol education program (NCEP) expert panel on detection, evaluation, and treatment of high blood cholesterol in adults (adult treatment panel III) final report. Circulation. (2002) 106(25):3143–421. 10.1161/circ.106.25.3143 12485966

[66] Poznyak A Grechko AV Poggio P Myasoedova VA Alfieri V Orekhov AN. The diabetes mellitus-atherosclerosis connection: the role of lipid and glucose metabolism and chronic inflammation. Int J Mol Sci. (2020) 21(5):1835. 10.3390/ijms21051835 32155866 PMC7084712

[67] Genders TS Steyerberg EW Hunink MG Nieman K Galema TW Mollet NR Prediction model to estimate presence of coronary artery disease: retrospective pooled analysis of existing cohorts. Br Med J. (2012) 344:e3485. 10.1136/bmj.e3485 22692650 PMC3374026

[68] Muhammad LJ Al-Shourbaji I Haruna AA Mohammed IA Ahmad A Jibrin MB. Machine learning predictive models for coronary artery disease. SN Comput Sci. (2021) 2(5):350. 10.1007/s42979-021-00731-4 34179828 PMC8218284

[69] Wang C Zhao Y Jin B Gan X Liang B Xiang Y Development and validation of a predictive model for coronary artery disease using machine learning. Front Cardiovasc Med. (2021) 8:614204. 10.3389/fcvm.2021.614204 33634169 PMC7902072

[70] Bogers RP Bemelmans WJ Hoogenveen RT Boshuizen HC Woodward M Knekt P Association of overweight with increased risk of coronary heart disease partly independent of blood pressure and cholesterol levels: a meta-analysis of 21 cohort studies including more than 300 000 persons. Arch Intern Med. (2007) 167(16):1720–8. 10.1001/archinte.167.16.1720 17846390

[71] Jung UJ Choi MS. Obesity and its metabolic complications: the role of adipokines and the relationship between obesity, inflammation, insulin resistance, dyslipidemia and nonalcoholic fatty liver disease. Int J Mol Sci. (2014) 15(4):6184–223. 10.3390/ijms15046184 24733068 PMC4013623

[72] Ohashi N Yamamoto H Horiguchi J Kitagawa T Kunita E Utsunomiya H Association between visceral adipose tissue area and coronary plaque morphology assessed by CT angiography. JACC Cardiovasc Imaging. (2010) 3(9):908–17. 10.1016/j.jcmg.2010.06.014 20846624

[73] Volpe M Gallo G. Obesity and cardiovascular disease: an executive document on pathophysiological and clinical links promoted by the Italian society of cardiovascular prevention (SIPREC). Front Cardiovasc Med. (2023) 10:1136340. 10.3389/fcvm.2023.1136340 36993998 PMC10040794

[74] Steverson A Pawlowski AE Schneider D Nannapaneni P Sanders J Achenbach CJ Associations of HIV-related factors with adjudicated heart failure (HF) in an electronic cohort of HIV-infected (HIV+) persons. J Am Coll Cardiol. (2017) 69(11):740. 10.1016/S0735-1097(17)34129-3

[75] Yen YF Ko MC Yen MY Hu BS Wang TH Chuang PH Human immunodeficiency virus increases the risk of incident heart failure. J Acquir Immune Defic Syndr. (2019) 80(3):255–63. 10.1097/QAI.0000000000001917 30531301

[76] Messner B Bernhard D. Smoking and cardiovascular disease: mechanisms of endothelial dysfunction and early atherogenesis. Arterioscler Thromb Vasc Biol. (2014) 34(3):509–15. 10.1161/ATVBAHA.113.300156 24554606

[77] McEvoy JW Nasir K DeFilippis AP Lima JA Bluemke DA Hundley WG Relationship of cigarette smoking with inflammation and subclinical vascular disease: the multi-ethnic study of atherosclerosis. Arterioscler Thromb Vasc Biol. (2015) 35(4):1002–10. 10.1161/ATVBAHA.114.304960 25745060 PMC4484586

[78] Morris PB Ference BA Jahangir E Feldman DN Ryan JJ Bahrami H Cardiovascular effects of exposure to cigarette smoke and electronic cigarettes: clinical perspectives from the prevention of cardiovascular disease section leadership council and early career councils of the American College of Cardiology. J Am Coll Cardiol. (2015) 66(12):1378–91. 10.1016/j.jacc.2015.07.037 26383726

[79] Kamimura D Cain LR Mentz RJ White WB Blaha MJ DeFilippis AP Cigarette smoking and incident coronary artery disease: insights from the Jackson heart study. Circulation. (2018) 137(24):2572–82. 10.1161/CIRCULATIONAHA.117.031912 29661945 PMC6085757

[80] Hall ME Wang W Okhomina V Agarwal M Hall JE Dreisbach AW Cigarette smoking and chronic kidney disease in African Americans in the Jackson heart study. J Am Heart Assoc. (2016) 5(6):e003280. 10.1161/JAHA.116.003280 27225196 PMC4937270

[81] Mu H Chai H Lin PH Yao Q Chen C. Current update on HIV-associated vascular disease and endothelial dysfunction. World J Surg. (2007) 31(4):632–43. 10.1007/s00268-006-0730-0 17372667

[82] Dau B Holodniy M. The relationship between HIV infection and cardiovascular disease. Curr Cardiol Rev. (2008) 4(3):203–18. 10.2174/157340308785160589 19936197 PMC2780822

[83] Phillips AN Carr A Neuhaus J Visnegarwala F Prineas R Burman WJ Interruption of antiretroviral therapy and risk of cardiovascular disease in persons with HIV-1 infection: exploratory analyses from the SMART trial. Antivir Ther. (2008) 13(2):177–87. 10.1177/135965350801300215 18505169

[84] Baker JV Sharma S Achhra AC Bernardino JI Bogner JR Duprez D Changes in cardiovascular disease risk factors with immediate versus deferred antiretroviral therapy initiation among HIV-positive participants in the START (strategic timing of antiretroviral treatment) trial. J Am Heart Assoc. (2017) 6(5):e004987. 10.1161/JAHA.116.004987 28533305 PMC5524070

[85] Obeagu EI. Influence of cytokines on the recovery trajectory of HIV patients on antiretroviral therapy: a review. Medicine (Baltimore). (2025) 104(1):e41222. 10.1097/MD.0000000000041222 40184131 PMC11709159

[86] Starikova EA Mammedova JT Rubinstein AA Sokolov AV Kudryavtsev IV. Activation of the coagulation cascade as a universal danger sign. Curr Issues Mol Biol. (2025) 47(2):108. 10.3390/cimb47020108 39996829 PMC11854423

[87] Funderburg NT Mayne E Sieg SF Asaad R Jiang W Kalinowska M Increased tissue factor expression on circulating monocytes in chronic HIV infection: relationship to *in vivo* coagulation and immune activation. Blood. (2010) 115(2):161–7. 10.1182/blood-2009-03-210179 19828697 PMC2808148

[88] Flint OP Noor MA Hruz PW Hylemon PB Yarasheski K Kotler DP The role of protease inhibitors in the pathogenesis of HIV-associated lipodystrophy: cellular mechanisms and clinical implications. Toxicol Pathol. (2009) 37(1):65–77. 10.1177/0192623308327119 19171928 PMC3170409

[89] den Boer MA Berbée JF Reiss P van der Valk M Voshol PJ Kuipers F Ritonavir impairs lipoprotein lipase-mediated lipolysis and decreases uptake of fatty acids in adipose tissue. Arterioscler Thromb Vasc Biol. (2006) 26(1):124–9. 10.1161/01.ATV.0000194073.87647.10 16269669

[90] Tran H Robinson S Mikhailenko I Strickland DK. Modulation of the LDL receptor and LRP levels by HIV protease inhibitors. J Lipid Res. (2003) 44(10):1859–69. 10.1194/jlr.M200487-JLR200 12837856

[91] Anuurad E Bremer A Berglund L. HIV Protease inhibitors and obesity. Curr Opin Endocrinol Diabetes Obes. (2010) 17(5):478–85. 10.1097/MED.0b013e32833dde87 20717021 PMC3076638

[92] Hresko RC Hruz PW. HIV Protease inhibitors act as competitive inhibitors of the cytoplasmic glucose binding site of GLUTs with differing affinities for GLUT1 and GLUT4. PLoS One. (2011) 6(9):e25237. 10.1371/journal.pone.0025237 21966466 PMC3179492

[93] Sherman J Dyar C McDaniel J Funderburg NT Rose KM Gorr M Sexual minorities are at elevated risk of cardiovascular disease from a younger age than heterosexuals. J Behav Med. (2022) 45(4):571–9. 10.1007/s10865-021-00269-z 35034218 PMC9287494

[94] Stacey L. An updated data portrait of heterosexual, gay/lesbian, bisexual, and other sexual minorities in the United States. Soc Curr. (2024) 11(5):383–400. 10.1177/23294965241260057

[95] van Kampen SC Lee WE Fornasiero M Husk K. The proportion of the population of England that self-identifies as lesbian, gay or bisexual: producing modelled estimates based on national social surveys. BMC Res Notes. (2017) 10(1):594. 10.1186/s13104-017-2921-1 29132439 PMC5683336

[96] Bonomo JA Luo K Ramallo JA. LGBTQ+ cardiovascular health equity: a brief review. Front Cardiovasc Med. (2024) 11:1350603. 10.3389/fcvm.2024.1350603 38510198 PMC10951381

[97] Caceres BA Streed CG Jr Corliss HL Lloyd-Jones DM Matthews PA Mukherjee M Assessing and addressing cardiovascular health in LGBTQ adults: a scientific statement from the American Heart Association. Circulation. (2020) 142(19):e321–32. 10.1161/CIR.0000000000000914 33028085 PMC8212867

[98] Maqsood MH Levine GN Kleiman ND Hasdai D Uretsky BF Birnbaum Y. Do we still need aspirin in coronary artery disease? J Clin Med. (2023) 12(24):7534. 10.3390/jcm12247534 38137601 PMC10743767

[99] Cholesterol Treatment Trialists’ (CTT) Collaboration. Efficacy and safety of more intensive lowering of LDL cholesterol: a meta-analysis of data from 170,000 participants in 26 randomised trials. Lancet. (2010) 376(9753):1670–81. 10.1016/S0140-6736(10)61350-5 21067804 PMC2988224

[100] Wilson PW Polonsky TS Miedema MD Khera A Kosinski AS Kuvin JT. Systematic review for the 2018 AHA/ACC/AACVPR/AAPA/ABC/ACPM/ADA/AGS/APhA/ASPC/NLA/PCNA guideline on the management of blood cholesterol: a report of the American College of Cardiology/American Heart Association task force on clinical practice guidelines. J Am Coll Cardiol. (2019) 73(24):3210–27. 10.1161/CIR.0000000000000626 30423394

[101] Ridker PM Danielson E Fonseca FA Genest J Gotto AM Jr Kastelein JJ Rosuvastatin to prevent vascular events in men and women with elevated C-reactive protein. N Engl J Med. (2008) 359(21):2195–207. 10.1056/NEJMoa0807646 18997196

[102] World Health Organization (WHO). Global status Report on Noncommunicable Diseases 2014. Geneva: World Health Organization (2014).

[103] Antiretroviral Therapy Cohort Collaboration. Life expectancy of individuals on combination antiretroviral therapy in high-income countries: a collaborative analysis of 14 cohort studies. Lancet. (2008) 372(9635):293–9. 10.1016/S0140-6736(08)61113-7 18657708 PMC3130543

[104] Fowler G. The strategy of preventive medicine. Br J Gen Pract. (1992) 42(364):495. Available online at: https://pmc.ncbi.nlm.nih.gov/articles/PMC1372287/

[105] GBD. DALYs and HALE collaborators. Global, regional, and national disability-adjusted life years (DALYs) for 306 diseases and injuries and healthy life expectancy (HALE) for 188 countries, 1990–2013: quantifying the epidemiological transition. Lancet. (2015) 386(10009):2145–91. 10.1016/S0140-6736(15)61340-X 26321261 PMC4673910

[106] Friis-Møller N Weber R Reiss P Thiébaut R Kirk O d’Arminio Monforte A Cardiovascular disease risk factors in HIV patients—association with antiretroviral therapy. Results from the DAD study. AIDS. (2003) 17(8):1179–93. 10.1097/01.aids.0000060358.78202.c1 12819520

[107] Naar-King S Parsons JT Johnson AM. Motivational interviewing targeting risk reduction for people with HIV: a systematic review. Curr HIV/AIDS Rep. (2012) 9(4):335–43. 10.1007/s11904-012-0132-x 22890780

