# Supplementary material for: Characterization and risk stratification of coronary artery disease in people living with HIV: a global systematic review
Source: Front Cardiovasc Med. 2025 Aug 12;12:1586019. doi: 10.3389/fcvm.2025.1586019 (PMC12378808; doi:10.3389/fcvm.2025.1586019)
Supplement: Supplementary file 1 [file Datasheet1.docx]

Supplementary File 1 (S1): Theoretical frameworks for risk stratification of CAD among PLWH

This appendix elaborates on the theoretical frameworks underpinning the review’s methodology for analyzing coronary artery disease (CAD) risk factors in people living with HIV (PLWH). Four models informed the conceptual approach (S1 Figure 1), each contributing strengths for stratifying multifactorial and context-dependent risk relationships.

# Principle 1: Broad-spectrum sampling of risk factors

This study comprehensively sampled all risk factors of HIV-related stroke, irrespective of time, setting, or age. Six major databases were searched to ensure that all potential risk factors of HIV-related stroke were included. The epidemiological triangle [1] guided this process allowing for the identification of HIV-specific (e.g., (cluster of differentiation-4 (CD4) count, viral load, use of antiretroviral therapy [ART]), cardiovascular (hypertension, diabetes, smoking, dyslipidemia, obesity etc.), intrinsic (age, sex, education), and extrinsic (income, employment status, access to healthcare) variables. This framework allows for the structured categorization of exposures but does not assess causality. Additional models are needed for deeper analysis.

# Principle 2: Non-communicable diseases (NCDs) result from multiple interacting causes

# The epidemiological triangle recognizes disease as an outcome of agent-host-environment interactions, but cannot quantify each factor’s contribution in polygenic NCDs. For effective prevention, contributing factors must be prioritized by clinical and public health relevance. This review applies Bradford Hill's criteria [2], Rothman’s causal pie [3], and Nweke’s hypotheses [4] to infer causality and stratify causative (risk) factors. While the strength of association is commonly used to stratify risk factors, it is insufficient alone [2]. Given the absence of robust quantitative models for estimating dimensions such as temporality, consistency, biological gradients, and specificity, this review incorporates emerging conceptual frameworks to address these limitations.

# Principle 3: Objective application of causal frameworks

Hill's and Rothman's models are applied with methodological support from emerging hypotheses [5-8, 9], which challenge the adequacy of statistical significance (magnitude of association) alone for attributing causality. These hypotheses introduce the cumulative risk index and emphasize specificity in exposure-outcome associations. Nweke and colleagues extended these frameworks to develop a causality estimation model [6], integrating Bradford Hill’s and Rothman’s principles to assess complex exposure-outcome relationships.

# Theoretical models

## Epidemiological triangle

Conceptualizes disease, CAD, as an outcome of interactions between:

- *Agent*: HIV and ART, particularly their inflammatory and metabolic effects.
- *Host*: Intrinsic characteristics such as age, genetics, immune status, and comorbidities such as hypertension or diabetes.
- *Environment*: Social determinants of health, healthcare access, geographic location, and lifestyle behaviors.

## Bradford Hill’s criteria for causality

A heuristic for evaluating causal associations using nine principles: strength, consistency, specificity, temporality, biological gradient, plausibility, coherence, experiment, and analogy.

- *Strength*: Larger associations are more likely to be causal.
- *Consistency*: Repeated observations in different settings and periods.
- *Temporality*: Cause precedes effect.
- *Biological gradient*: Dose-response relationship.
- *Plausibility & coherence*: Aligns with biological mechanisms.

Limitations include subjectivity and limited capacity to address confounding and complex interactions.

## Rothman’s Causal Pie Model

Posits that disease arises from combinations of component causes forming a sufficient cause (complete pie).

*Necessary causes* (must be present)

- *Component causes* (contribute but not sufficient alone)
- *Sufficient cause combinations* (complete causal paths)

This model supports hierarchical classification and the analysis of synergistic interactions.

## Nweke’s cumulative risk index (CRI) framework

Extends Rothman and Hill’s models with new stratification criteria:

- *Predictive consistency (risk weight)*: Reproducible associations across contexts.
- *Irreversibility*: The risk factor induces permanent physiological or structural changes; it cannot be protective.
- *Geotemporal trend*: Measures global and temporal spread of risk factors; favors universally prevalent risk factors.
- *Scoring system:* Causality index (CI) and risk weight (Rw) are assigned based on temporality, plausibility, and public health priority.

Categories:

- *Necessary causes*: High CI and Rw (e.g., hypertension)
- *Synergistic/component causes*: Moderate CI and Rw (e.g., unsuppressed viral load)
- *Low-priority/unstable predictors*: Low CI and Rw, often context-specific

This framework enables evidence synthesis even in the absence of individual participant data and supports prioritization in resource-limited settings.

# Conclusion

These models, used in a complementary manner, enhance the interpretive depth and contextual validity of this review. Their integration provides a robust conceptual foundation for evidence classification and CAD risk stratification in PLWH.


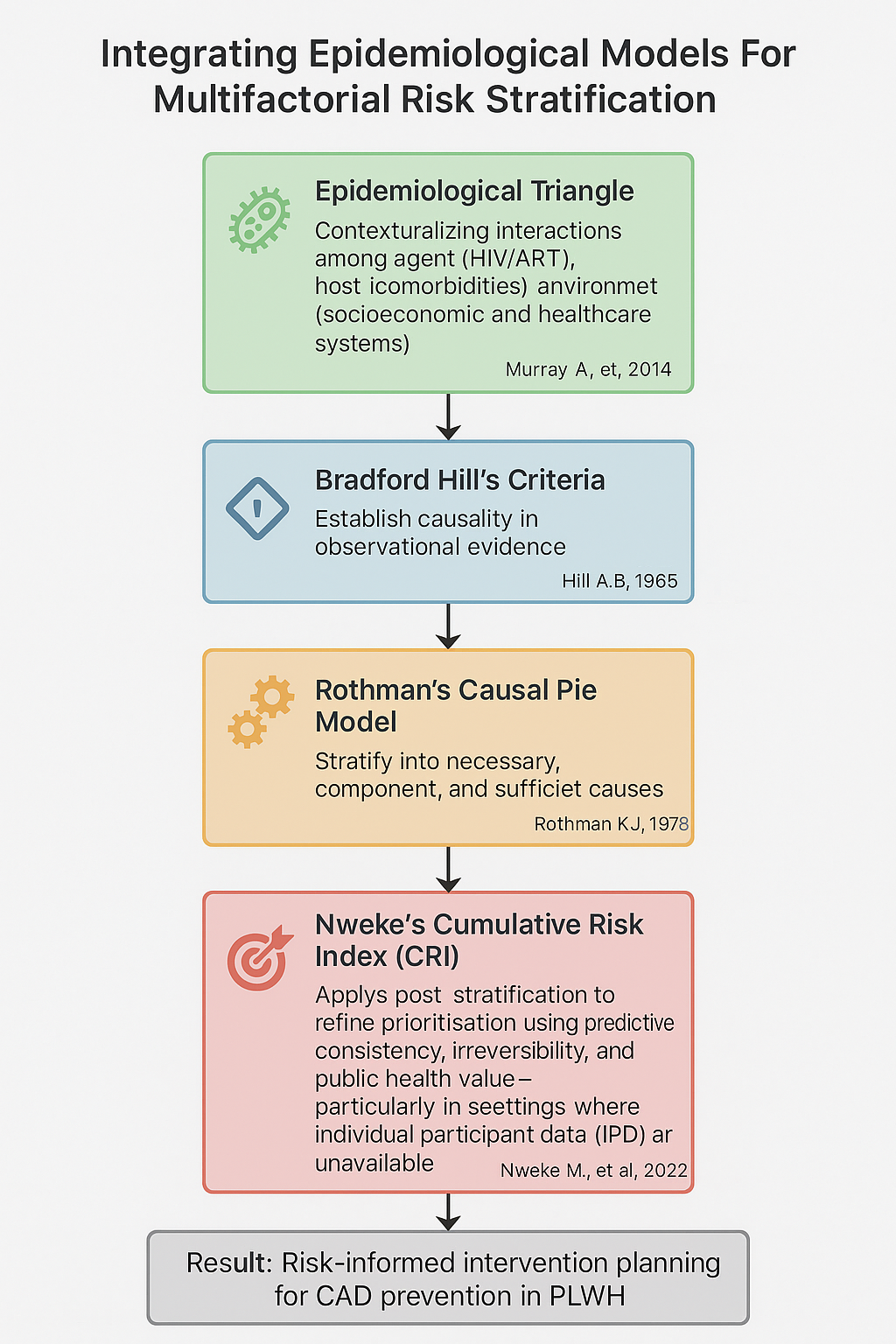


**S1 Figure 1: Flowchart illustrating the application of integrated theoretical models for CAD risk stratification in PLWH**

References:

1. Murray A, Huang MJ, Hardnett F, Sutton MY. Strengthening HIV knowledge and awareness among undergraduate students at historically black colleges and universities. Journal of Health Disparities Research and Practice. 2014;7(4):4.
2. Hill AB. The environment and disease: association or causation? *Proc R Soc Med.* 1965;58(5):295–300.
3. Rothman KJ. Causes. American journal of epidemiology. 1976 Dec 1;104(6):587-92.
4. Nweke M, Ukwuoma M, Adiuku-Brown AC, Ugwu P, Nseka E. Characterization and stratification of the correlates of postpartum depression in sub-Saharan Africa: A systematic review with meta-analysis. Women's Health. 2022 Aug;18:17455057221118773.
5. Nweke M, van Vuuren M, Bester K, Maritz A, van Vuuren L, Vilakazi Y, Dlamini A, Ncedani A, Mostert K. Association between socio-demographic and injury factors, and physical activity behaviour in people with spinal cord injury: a theory-informed systematic review and meta-analysis. BMC Sports Science, Medicine and Rehabilitation. 2025;17:179.
6. Nweke M, Mshunqane N. Characterization and stratification of risk factors of stroke in people living with HIV: A theory-informed systematic review. BMC Cardiovascular Disorders. 2025;25(1):405.
7. Nweke M, Oyirinnaya P, Nwoha P, Mitha SB, Mshunqane N, Govender N, Ukwuoma M, Ibeneme SC. Development of a high-performing, cost-effective and inclusive Afrocentric predictive model for stroke: a meta-analysis approach. BMC neurology. 2025 Jul 7;25(1):282.
8. Nwagha T, Nweke M. Stratification of risk factors of lung cancer-associated venous thromboembolism and determining the critical point for preemptive intervention: A systematic review with meta-analysis. Clinical Medicine Insights: Oncology. 2023 Jun;17:11795549231175221.
9. Ibeneme SC, Odoh E, Martins N, Ibeneme GC. Developing an HIV-specific falls risk prediction model with a novel clinical index: a systematic review and meta-analysis method. BMC Infectious Diseases. 2024;24(1):1402.
