# Supplementary material for: Characterization and risk stratification of coronary artery disease in people living with HIV: a global systematic review
Source: Front Cardiovasc Med. 2025 Aug 12;12:1586019. doi: 10.3389/fcvm.2025.1586019 (PMC12378808; doi:10.3389/fcvm.2025.1586019)
Supplement: Supplementary file 2 [file Datasheet2.docx]

Supplementary File 2: Epidemiological metrics and worked examples

1. **Geotemporal trend (GTT)**

GTT measures the universality of a risk factor across geographic regions and time periods. The prevalence and impact (“virulence”) of risk factors often vary by location and time, making it essential to assess both spatial and temporal coverage when prioritizing risk factors for global health initiatives. This ensures that risk factors with broad applicability and public health relevance are prioritized.

In this study, data were drawn from six continents: Europe, North America, Australia, South America, Asia, and Africa. Geographical coverage was defined as the ratio of continents represented in the synthesis of a specific risk factor to the total number of continents (six). Temporal coverage referred to the representation of risk factors across three defined decades and was calculated similarly.

GTT is a product of geographical and temporal coverage. A GTT of ≥0.6 is considered acceptable, indicating representation in at least three continents and two decades. The final GTT scores were adjusted for publication bias and risk responsiveness, which is inversely related to the number of studies contributing to the synthesis. In this study, since most of the risk factors did not meet the 0.6 cut-off, we used 0.5. A GTT of 0.5 means that the evidence underlying the risk factor was drawn from three of the six continents, and approximately two out of the three periods. The use of lower GTT cut-off constitutes a limitation regarding the generalizability of the emergent risk factors and models.

Example:

Hypertension was reported in five of six continents and across all three decades.

Geographical coverage = 5/6 = 0.83

Temporal coverage = 3/3 = 1.00

GTT = 0.83 x 1.00 = 0.83

Adjusted GTT = (GTT + Ri)/2 = 0.83 +0.91/2 = 0.87

Where Ri is a derivative of ΣF (total number of articles involved in the meta-synthesis).

## Application

Risk factors with high GTT scores and significant risk attribution (i.e., classified as necessary or component causes) are prioritized in global screening models and primary prevention strategies.

1. **Risk stratification using risk weight, causality index, and public health priority score**

Coronary artery disease (CAD) risk factors were stratified using three metrics, namely risk weight (Rw), causality index (CI), and public health priority (PHP) score.

## Risk weight and risk responsiveness (consistency)

Rw, also termed the N-factor (after M Nweke), quantifies predictive consistency, incorporating the strength and stability of association. It is based on two of Hill’s criteria: strength of association (typically measured by the odds ratio [OR]) and consistency (Ri). Only significant (p <0.05) ORs are considered.

Formula: Rw = OR x Ri

Where Ri = F/ΣF and F = number of studies reporting statistical significance for the risk factor, and ΣF = total number of studies included in the synthesis

## Example: CAD and hypertension

Eleven studies assessed the association between CAD and hypertension; and 10 reported hypertension as a significant risk factor. The pooled OR for hypertension was 4.90 (p <0.05). Therefore, Ri = 10/11 = 0.91. The Rw = 4.90 x 0.91 = 4.50.

Risk factors were stratified into three classes based on Rw quartiles:

- Third class: Q1–Q2 (lowest 50%)
- Second class: Q2–Q3 (50^th^ to 75^th^ percentile)
- First class: Q3–Q4 (highest 25%)

In this study, the quartile thresholds were Q1 = 0.9, Q2 = 1.32, and Q3 = 2.2. Rw was used to determine the critical risk threshold (CRT), which marks the point where risk accumulation predicts disease onset (Figure 1). For primary prevention, the CRT was set at Rw = 14.8 (75^th^ percentile); for secondary prevention, at Rw = 8.0 (50^th^ percentile).

The CRT represents the cumulative Rw required to transition from subclinical to clinical disease, with “necessary causes” as required elements and “component causes” as synergistic contributors.


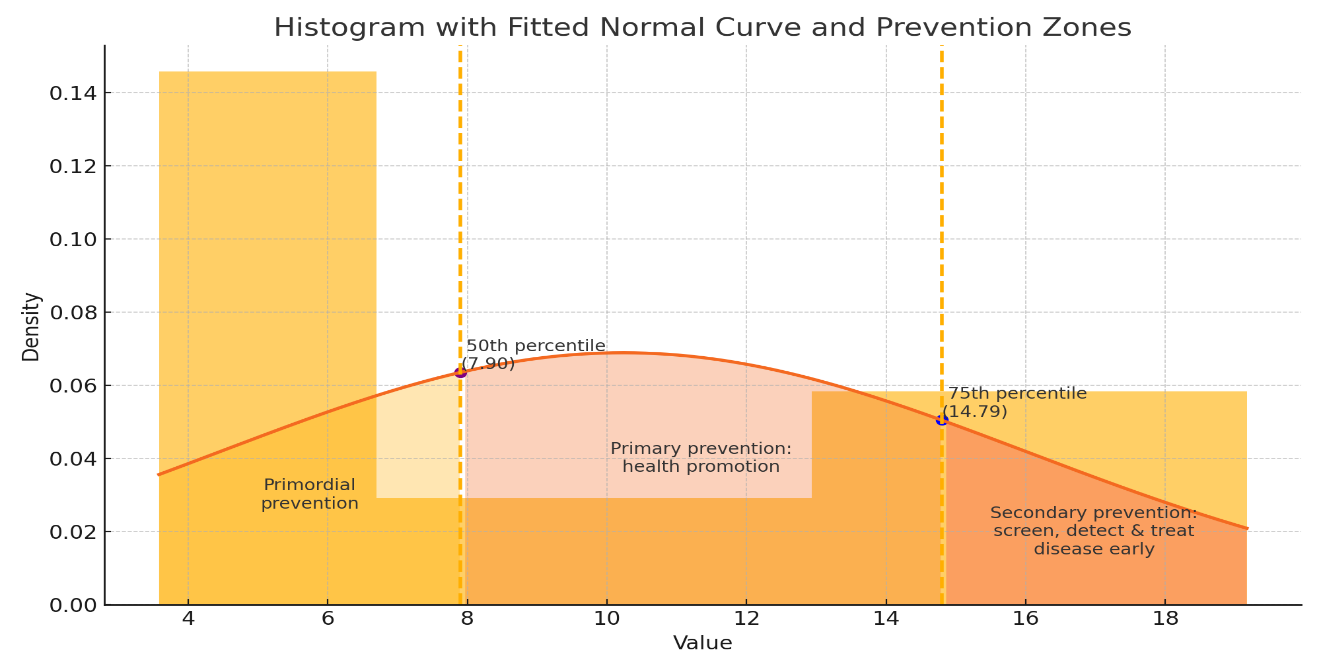


S2 Figure 1: Histogram showing the Rw-based CRT for different levels of prevention

**Example**: In this study, we assessed 11 CAD risk factors: age, hypertension, family history of cardiovascular disease (CVD), history of AIDs-defining illness, dyslipidemia, obesity, diabetes, smoking, viral load, sexual orientation, and use of antiretroviral therapy (protease inhibitors, abacavir, nucleoside reverse transcriptase inhibitors [NRTI] and non-RTI).

Combinations of these factors that yield a cumulative Rw ≥14.8 represent valid causal models. Over 130 combinations met this threshold (S2 Table 1). Age and hypertension represented necessary causes and were present in all valid combinations. The remaining factors were identified as component causes.

To ensure representativeness, high performance and ease of assessment, we preferred parsimonious models with high summative Rw and GTT, provided that the risk factor met the clinically meaningful important difference (CMID) criterion. The hypothetical definition of CMID remains an increase in risk by at least approximately 0.5 (50%). All the risk factors except viral load met the CMID definition.

The most parsimonious model (Rw ≥14.8, net GTT = 4.55) included age (Rw = 3.58), hypertension (Rw = 4.50), family history of CVD (Rw = 2.24), dyslipidemia (Rw = 2.15), overweight/obesity (Rw = 1.36), and diabetes (Rw = 1.32) as risk factors. The most cost-effective model (Rw = 15.17, net GTT = 4.90) included age (Rw = 3.58), hypertension (Rw = 4.50), family history of CVD (Rw = 2.24), overweight/obesity (Rw = 1.36), history of AIDS-defining illness (Rw = 0.97), ART exposure (Rw = 1.06), sexual orientation (Rw = 0.62), and smoking (Rw = 0.84) as risk factors.

**S2 Table 1: Coronary artery disease (CAD) risk factors and checklist combinations**

| **Model number** | **Risk factors (Rw)** | **Summative risk weight (Rw)** | **Net geotemporal trend (GTT)** |
| --- | --- | --- | --- |
| 1 | Age (3.58), Hypertension (4.50), Family history of CVD (2.24), Dyslipidemia (2.15), Overweight/obesity (1.36), History of AIDS‑defining illness (0.97) | 14.80 | 4.19 |
| 2 | Age (3.58), Hypertension (4.50), Family history of CVD (2.24), Dyslipidemia (2.15), Diabetes (1.32), History of AIDS‑defining illness (0.97) | 14.76 | 4.16 |
| 3 | Age (3.58), Hypertension (4.50), Family history of CVD (2.24), Dyslipidemia (2.15), History of AIDS‑defining illness (0.97), Viral load (0.53), Smoking (0.84) | 14.81 | 4.85 |
| 4 | Age (3.58), Hypertension (4.50), Family history of CVD (2.24), Overweight/obesity (1.36), Diabetes (1.32), History of AIDS‑defining illness (0.97), Smoking (0.84) | 14.81 | 4.61 |
| 5 | Age (3.58), Hypertension (4.50), Dyslipidemia (2.15), Overweight/obesity (1.36), Diabetes (1.32), ART exposure (1.06), Smoking (0.84) | 14.81 | 4.51 |
| 6 | Age (3.58), Hypertension (4.50), Family history of CVD (2.24), Diabetes (1.32), History of AIDS‑defining illness (0.97), Viral load (0.53), ART exposure (1.06), Sexual orientation (0.62) | 14.82 | 4.9 |
| 7 | Age (3.58), Hypertension (4.50), Dyslipidemia (2.15), Overweight/obesity (1.36), History of AIDS‑defining illness (0.97), Viral load (0.53), ART exposure (1.06), Sexual orientation (0.62) | 14.77 | 4.99 |
| 8 | Age (3.58), Hypertension (4.50), Overweight/obesity (1.36), Diabetes (1.32), History of AIDS‑defining illness (0.97), Viral load (0.53), ART exposure (1.06), Sexual orientation (0.62), Smoking (0.84) | 14.78 | 5.41 |
| 9 (Most parsimonious) | Age (3.58), Hypertension (4.50), Family history of CVD (2.24), Dyslipidemia (2.15), Overweight/obesity (1.36), Diabetes (1.32) | 15.15 | 4.05 |
| 10 (Most cost-effective) | Age (3.58), Hypertension (4.50), Family history of CVD (2.24), Overweight/obesity (1.36), History of AIDS-defining illness (0.97), ART exposure (1.06), Sexual orientation (0.62), Smoking (0.84) | 15.17 | 4.9 |

1. **Causality index and public health priority**

The CI incorporates four key criteria: strength of association, consistency (Ri), temporality, and biological gradient. Since temporality can only be determined via a cohort or experimental study, we defined temporality as the ratio of cohort studies to total studies included in the meta-synthesis. Temporality was excellent if the ratio ≥0.75 (value 3), fair is the ratio ranged between 0.74–0.5 (value 2), and poor if the ratio was <0.5 (value 1).

Strength of association was classified as 1 if the OR ≤ 2, 2 if the OR ranged from 2–4.4, and 3 if the OR was ≥4.5. Consistency (Ri) was deemed perfect if Ri = 1 (Value = 3), good within 0.8–0.9 (Value = 2), fair within 0.6–0.7 (Value = 1), and poor if Ri <0.6 (Value = 0). Biological gradient represented the irreversibility of the association. If all the significant findings were in the same direction, then the biological gradient = 1, mixed findings = 0.

CI classification

First-class risk factors: CI 7–10

Second-class risk factor: CI 5–6

Third-class risk factors: CI ≤4

Example: In this study, the association between hypertension and CAD was examined in 11 studies, of which three were cohort studies. The temporality coefficient = 3/11 = 0.27, scoring 1 point. The consistency (Ri) was 0.91 and rated as excellent (3). The biological gradient was rated as 1, as all the significant findings were in the same direction. The strength of association was 4.9 (p <0.05), and rated as 3. Therefore, the CI for hypertension = 3 + 3 + 1 + 1 = 8/10. Hypertension was thus classified as a first-class risk factor.

1. **Public health priority (PHP)**

Biobehavioral approaches to disease prevention target both behavioral and biological causes of disease. PHP frameworks integrate the CI score and the nature or stage of exposure. Exposures can be classified as behavioral factors and biological factors. Biological factors can be further categorized into non-disease-related (e.g., body mass index, viral load, CD4 count) and disease-related factors (e.g., diabetes, hypertension, dyslipidemia). Mass prevention strategies typically prioritize behavioral determinants, while targeted prevention focuses on biological determinants.

Regarding the nature or stage of the risk factor, behavioral factors (e.g., smoking, viral load), non-disease-related biological factors (e.g., viral load, CD4 count, female sex, advanced age), and prevalent and "virulent" chronic biological diseases factors (e.g., diabetes, hypertension, dyslipidemia, HCV) were assigned a PHP score of 5. Conversely, non-prevalent biological disease factors, such as liver cirrhosis, were ranked lower and assigned the value of 1. Behavioral factors within third-class risk categories were not prioritized.

Factors (e.g. ART use) that are essential for disease treatment must be treated with high priority, irrespective of the PHP score. Overall, factors were assigned top PHP status if they met two conditions: belonging to either the first-class or second-class risk categories and were classified as modifiable behavioral factors (e.g., smoking, viral load), biological non-disease factors (e.g., viral load, CD4 count, female sex, advanced age), or prevalent and *"virulent"* chronic biological diseases (e.g., diabetes, hypertension, dyslipidemia, HCV). A PHP ≥10: high priority, 7-8: moderate priority and ≤6: low priority.

**Application**

The CI is integral for estimating the PHP of a risk factor. In our study, hypertension was a modifiable (index score of 5) and first-class (CI = 7) risk factor. Therefore, the PHP was 12 out of 15, indicating a high priority.

**Integrating risk weight and public health priority**

While Rw specifies the risk zones for different levels of prevention, and determine the targets for secondary prevention, the PHP specifies the therapeutic target for primary prevention. In our study, critical risk factors (Rw= 14.8) included age, hypertension, family history of CVD, dyslipidemia, overweight/obesity, and diabetes. Regarding PHP, critical risk factors (Rw = 7.9) included hypertension, dyslipidemia, and obesity. For early detection and treatment, the CI prioritizes high cumulative risk. For risk reduction at the population level, the PHP model emphasizes modifiable and prevalent exposures.
