# Supplementary material for: Characterization and risk stratification of coronary artery disease in people living with HIV: a global systematic review
Source: Front Cardiovasc Med. 2025 Aug 12;12:1586019. doi: 10.3389/fcvm.2025.1586019 (PMC12378808; doi:10.3389/fcvm.2025.1586019)
Supplement: Supplementary file 3 [file Datasheet3.docx]

Supplementary 3: JBI Critical Appraisal Summary

*Domains of bias assessment*

The risk of bias assessment covered the following key methodological domains:

1. Selection Bias: Evaluated whether study participants were adequately defined, representative of the target population, and selected in a manner that minimized bias.
2. Information Bias: Examined the accuracy and reliability of exposure and outcome measurements, including whether standardized or validated tools were used.
3. Confounding Bias: Assessed whether the studies appropriately accounted for confounders through study design or statistical adjustments.
4. Follow-up and attrition bias: The adequacy of follow-up in cohort studies, particularly whether losses to follow-up were reported and managed appropriately.
5. Reporting Bias: Determined whether selective outcome reporting occurred, ensuring that all relevant risk factors were presented transparently.

Supplementary 3 Table 1: JBI case–control critical appraisal summary

| Study | D1 | D2 | D3 | D4 | D5 | D6 | D7 | D8 | D9 | D10 | Score | Interpretation |
| --- | --- | --- | --- | --- | --- | --- | --- | --- | --- | --- | --- | --- |
| Berquist et al. 2017 | Y | Y | Y | Y | Y | Y | Y | Y | Y | Y | 10/10 | Low risk of bias |
| Bucher et al. 2011 | Y | Y | Y | Y | Y | Y | Y | Y | Y | Y | 10/10 | Low risk of bias |
| Egaña‑Gorroño et al. 2012 | Y | N | Y | Y | Y | Y | Y | Y | Y | Y | 9/10 | Moderate risk of bias |
| Engel et al. 2021 | Y | Y | Y | Y | Y | Y | Y | Y | Y | Y | 10/10 | Low risk of bias |
| Mushin et al. 2023 | Y | Y | Y | Y | Y | Y | Y | Y | Y | Y | 10/10 | Low risk of bias |
| Trevillyan et al. 2017 | Y | Y | Y | Y | Y | Y | Y | Y | Y | Y | 10/10 | Low risk of bias |
| Trevillyan et al. 2013 | Y | N | Y | Y | Y | Y | Y | Y | Y | Y | 9/10 | Moderate risk of bias |

U: Unclear, D1: Comparability of groups, D2: Matching, D3: Same selection criteria, D4: Valid exposure measurement, D5: Exposure period, D6: Identification of confounders, D7: Strategies for confounding, D8: Valid outcome measurement, D9: Same measurement method, D10: Appropriate analysis

Supplementary 3 Table 2: JBI case–control critical appraisal summary

| Study (year) | D1 | D2 | D3 | D4 | D5 | D6 | D7 | D8 | D9 | D10 | D11 | Score | Interpretation |
| --- | --- | --- | --- | --- | --- | --- | --- | --- | --- | --- | --- | --- | --- |
| Chammartin et al. 2022 | Y | Y | Y | Y | Y | Y | Y | Y | U | N | Y | 9/11 | Low risk of bias |
| Dale et al. 2017 | Y | Y | Y | Y | N | Y | Y | N | N | N | Y | 7/11 | Moderate risk |
| Escaut et al. 2003 | Y | Y | Y | Y | N | Y | Y | Y | U | N | N | 7/11 | Moderate risk |
| Freiberg et al. 2011 | Y | Y | Y | Y | Y | Y | Y | Y | U | N | Y | 9/11 | Low risk of bias |
| Hadigan et al. 2003 | Y | Y | Y | Y | Y | Y | Y | Y | U | N | Y | 9/11 | Low risk of bias |
| Kaplan et al. 2007 | Y | Y | Y | Y | Y | Y | Y | Y | U | N | Y | 9/11 | Low risk of bias |
| Lai et al. 2008 | Y | Y | Y | Y | Y | Y | Y | Y | U | N | Y | 9/11 | Low risk of bias |
| Longenecker et al. 2022 | Y | Y | Y | Y | Y | Y | Y | N | N | N | Y | 8/11 | Moderate risk |
| Urina-Jassir et al. 2023 | Y | Y | Y | Y | Y | N | Y | N | N | N | Y | 7/11 | Moderate risk |
| Worm et al. 2009 | Y | Y | Y | Y | Y | Y | Y | Y | U | N | Y | 9/11 | Low risk of bias |
| May et al. 2007 | Y | Y | Y | Y | Y | Y | Y | Y | U | N | Y | 9/11 | Low risk of bias |

U:Unclear, D1: Groups similar, D2: Same exposure measurement, D3: Valid exposure measurement, D4: Confounders identified, D5: Confounders dealt with, D6: Outcome-free at baseline, D7: Valid outcome measurement, D8: Follow-up sufficient, D9: Follow-up complete, D10, Loss to follow-up addressed, D11; Appropriate analysis

Supplementary 3 Table 3: JBI cross-sectional critical appraisal summary

| Study | D1 | D2 | D3 | D4 | D5 | D6 | D7 | D8 | Score | Interpretation |
| --- | --- | --- | --- | --- | --- | --- | --- | --- | --- | --- |
| Trøseid et al. 2024 | Y | Y | Y | Y | Y | Y | Y | Y | 8/8 | Low risk of bias |
| Fuchs et al. 2013 | Y | Y | Y | Y | N | N | N | Y | 4/8 | Moderate |
| Kaplan et al. 2007 | Y | Y | Y | Y | Y | Y | Y | Y | 8/8 | Low risk of bias |
| Pullinger et al. 2010 | Y | Y | Y | Y | Y | N | Y | U | 6/8 | Moderate risk of bias |

U: unclear, D1: Criteria for inclusion in the sample clearly defined, D2: Study subjects and setting described in detail, D3: Exposure measured validly and reliably, D4: Condition (outcome) measured objectively using standard criteria, D5: Confounding factors identified, D6: Strategies to deal with confounding stated, D7: Confounders measured validly and reliably; D8: Appropriate statistical analysis used
